# Rac1 in parvalbumin neurons of the medial prefrontal cortex governs rapid forgetting of social memory

**DOI:** 10.1038/s41380-025-02963-9

**Published:** 2025-03-29

**Authors:** Meng Li, Shi-Zhe Wang, Ya-Bo Zhao, Xun Tang, Lin Xu, Hongsheng Wang, Qi-Xin Zhou

**Affiliations:** 1 Key Laboratory of Animal Models and Human Disease Mechanisms, and Laboratory of Learning and Memory, Kunming Institute of Zoology, Chinese Academy of Sciences, Kunming, 650223 China; 2 University of Chinese Academy of Sciences, Beijing, 101408 China; 3 Department of Traditional Chinese Medicine, Songjiang Research Institute, Shanghai Kay Laboratory of Emotions and Affective Disorders, Songjiang Hospital Affiliated to Shanghai Jiao Tong University School of Medicine, Shanghai, 201600 China; 4 Present Address: Department of Traditional Chinese Medicine, Songjiang Research Institute, Shanghai Kay Laboratory of Emotions and Affective Disorders, Songjiang Hospital Affiliated to Shanghai Jiao Tong University School of Medicine, Shanghai, 201600 China

**Keywords:** Neuroscience, Diagnostic markers

## Abstract

Social memory can undergo rapid forgetting at first according to the Ebbinghaus forgetting curve, for which the underlying mechanism remains entirely unknown. Here, we reported that rapid forgetting of social memory did not occur as indicated by social preference on stranger 2 (S2) over stranger 1 (S1) mouse, tested shortly after social interaction with S1. However, rapid forgetting of both social and object memories occurred as indicated by no social or object preference, respectively, when the constitutive active (CA) variant of Rac1 was knocked-in parvalbumin (PV) but not somatostatin (SST) neurons of the brain. Furthermore, rapid forgetting of only social memory occurred if this CA variant was knocked-in PV but not SST neurons of the medial prefrontal cortex (mPFC). By contrast, rapid forgetting of social memory was prevented by the dominant negative (DN) variant of Rac1 knocked-in PV neurons of the mPFC. Moreover, fiber photometry revealed that PV but not SST neurons of the mPFC generated dual calcium peaks to delineate each social interaction event. Thus, PV-specific Rac1 activity of the mPFC is both necessary and sufficient for controlling social behavior via rapid forgetting of social memory, providing a novel understanding of social behaviors under health and disease conditions.

## Introduction

Abnormal forgetting processes can cause problems in social memory, which plays an important role in the organization of daily social behaviors. A vast amount of short-term social memories is readily formed and only the most important ones are transformed into long-term memories but most of them are rapidly forgotten [1–3]. The Ebbinghaus forgetting curve indicates that memory goes through rapid forgetting immediately after learning and the forgetting speed slows after days. In particular, rapid forgetting of social memory can occur within a few seconds or minutes, temporally holding social information for organizing subsequent social behaviors such as communication, discussing, facial recognition, decision-making, etc. However, abnormal rapid forgetting of social memory can cause abnormal social behaviors. For example, social dysfunctions occur in post-traumatic stress disorder (PTSD) [4], which is possibly due to the lack of forgetting. Social dysfunctions also occur in Alzheimer’s disease [5] (AD), which is likely because of over forgetting.

There are many developments involving neural mechanisms for social memory and possibly it’s forgetting. Previous studies showed that mPFC, ACC, and CA1 involved in complex social behaviors and memory process. The medial prefrontal cortex (mPFC) has long been demonstrated to be critical for many cognitive functions including emotional regulation, self-referential thinking, prosocial behavior, and social memory [6, 7], which contribute to maintaining and navigating social bonds. The anterior cingulate cortex (ACC) participated in social signal processing, social pain, and social memory [8]. Neuroimaging studies have suggested that the ACC is active when human participants engage in social behaviors [9]. Many studies have indicated that hippocampal CA2 is particularly important for encoding social memory [10–12]. CA1 processes information passed from other hippocampal regions, such as CA2. Furthermore, our previous report suggests that long-term depression of hippocampal CA1 and social memory is abnormal in the mice knocked out *P-Rex1*, a risk gene of autism spectrum disorder (ASD) [13]. These studies have focused on glutamatergic neurons or mechanisms for social memory. For example, Rac1 activation in glutamatergic CA1 neurons promotes forgetting of long-term social memory [14]. However, Rac1 deactivation in dorsal hippocampal CA1 prevents the forgetting of long-term contextual fear memory [15]. In contrast, parvalbumin (PV) and somatostatin (SST) neurons belong to GABAergic system [16], and they are different in morphology, physiology, and activity patterns [17, 18], with innervations onto cell bodies and dendrites, respectively. PV neurons, expressing parvalbumin, are characterized by lower depolarization membrane potential, lower input resistance, and higher spike firing frequency [18]. These properties enable PV neurons to regulate other neurons rapidly. SST neurons, expressing somatostatin, have properties significantly different from PV neurons. Compared to PV neurons, SST neurons have higher depolarization membrane potential, higher input resistance, and slower firing kinetics [17]. To our best knowledge, no reports regarding PV and SST neurons may contribute to the rapid forgetting of social memory.

Previous developments have notably demonstrated that a small GTP protein Rac1 (Ras-related C3 botulinum toxin substrate 1) can serve as a molecular switch for controlling forgetting of certain types of memories [19–22], i.e., Rac1-GTP promotes forgetting but Rac1-GDP prevents forgetting [20]. Later studies have found that Rac1 has two genetic variants, a constitutively active (CA) and a dominant negative (DN) Rac1 variant to promote or prevent forgetting, respectively [20, 23]. Therefore, we have assumed that Rac1 activity in PV or SST neurons can govern rapid forgetting of social and/or object memories.

To address this question, we used Rac1(CA)-loxp mice and PV-Cre or SST-Cre mice, to generate Rac1(CA) knocked-in PV or SST neurons of the brain or the mPFC or ACC or hippocampal CA1. Social interaction and object recognition tests were then used to identify the effects of these manipulations on social/object memory and its rapid forgetting with 5-min as the interval between learning and memory tests. We reported here that Rac1 activity in PV neurons of the mPFC is both sufficient and necessary for rapid forgetting of social memory, as PV neurons of the mPFC are activated by each social interaction event. These results provide a novel understanding of social dysfunction in many brain disorders.

## Results

### Rapid forgetting of social recognition memory is enhanced by knocked-in Rac1(CA) into PV but not SST neurons of the brain

**Fig. 1:**
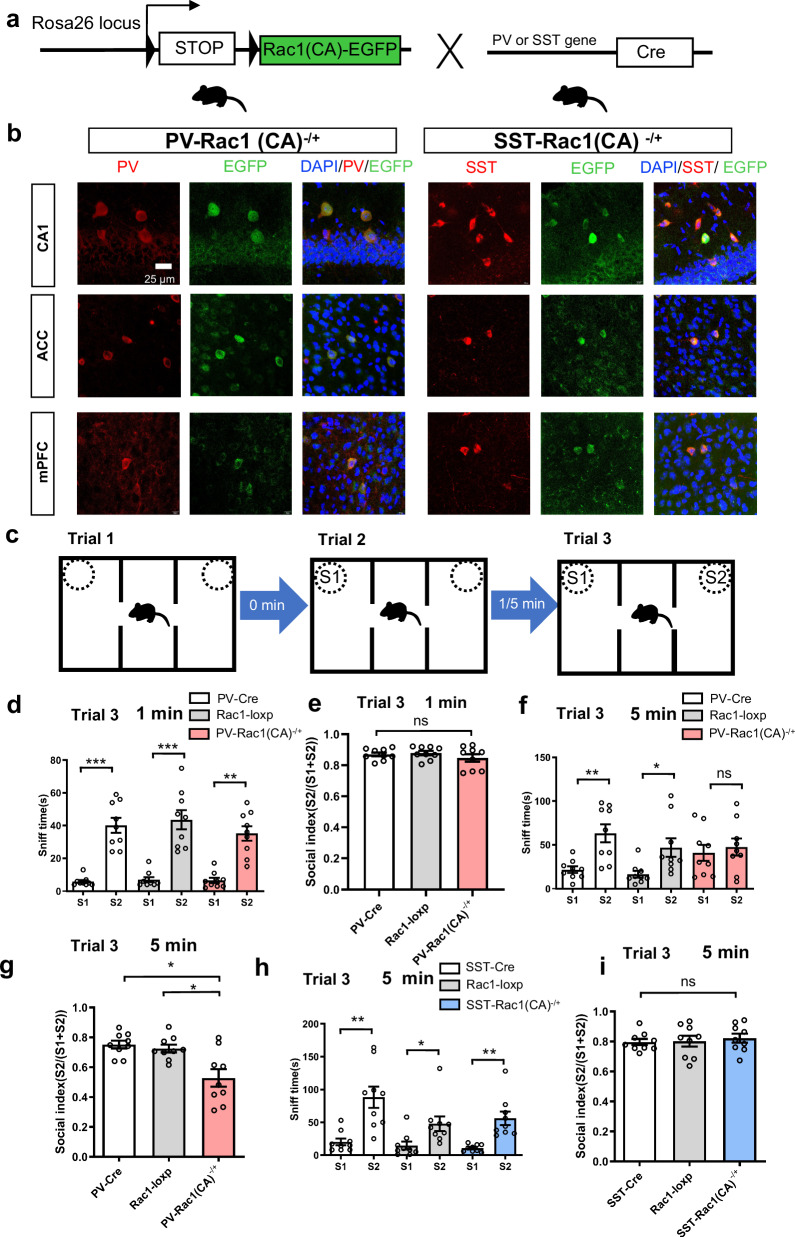
Rapid forgetting of social recognition memory is enhanced by Rac1(CA) in PV but not SST neurons of the brain. **a** Parvalbumin (PV)-Cre or somatostatin (SST)-Cre mice were crossed with Rac1-loxp mice containing a constitutive activation (CA) of Rac1 mutation, and only PV-Rac1(CA)^−/+^ or SST-Rac1(CA)^−/+^ mice were available for this study. **b** Representative images from the hippocampal CA1 region, anterior cingulate cortex (ACC), and medial prefrontal cortex (mPFC), in which PV or SST neurons were indicated by red signals. Rac1(CA) signals (green, immunostaining of GFP) were co-localized with PV or SST neurons in all these areas. **c** Experimental design for social memory using three-chamber sociability and social memory test, for which we designed the inter-trial intervals (ITIs) at either 1 or 5 min before trial 3. **d**, **e** When ITI was set at1 min, PV-Cre, Rac1-loxp and PV-Rac1(CA)^−/+^ mice went through social memory test. Social memory about the experience of stranger 1 (S1) mouse during trial 2 enabled social preference to stranger 2 (S2) mouse as indicated by more sniff time to S2 than to S1 mouse (n = 9/per group; S1 vs S2, ***p_1, 2_ < 0.001, **p = 0.002, Kruskal-Wallis test) and similar social index among the groups (n = 9/per group, *F*_(2,24)_ = 0.857, *p* = 0.437, one-way ANOVA). **f**, **g** When ITI was set at 5 min, social preference to S2 mouse as indicated by more sniff time to S2 than to S1 mouse was significant in controls (PV-Cre or Rac1-loxp) but not in PV-Rac1(CA)^−/+^ mice (n = 9/per group; *F*_(5,48)_ = 4.291, S1 vs S2, **p_1_ = 0.003, *p_2_ = 0.044, and *p* = 0.921, one-way ANOVA). Social index was higher in controls than in PV-Rac1(CA)^−/+^ mice (n = 9/per group; *F*_(2,14)_ = 9.339, *p_1_ = 0.01, *p_2_ = 0.02, Brown Forsythe ANOVA test), suggesting that constitutive activation of Rac1 in PV neurons caused rapid forgetting of social memory. **h**, **i** When ITI was set at 5 min, sniff time showed similar pattern in SST-Cre, Rac1-loxp, and SST-Rac1(CA)^−/+^ mice(n = 9/per group; S1 vs S2, **p_1_ = 0.004, *p_2_ = 0.016, and **p_3_ = 0.001, Kruskal-Wallis test) and social index was not different among groups (n = 9/per group; *F*_(2,24)_ = 0.211, *p* = 0.811, one-way ANOVA). All data showed as mean ± s.e.m.

We wanted to activate Rac1 in PV or SST neurons of the whole brain to see the effects on rapid forgetting. Two mouse lines with Rac1(CA) conditional knocked in PV or SST neurons were generated by using Rac1(CA)-loxp-EGFP mice crossed with PV-Cre or SST-Cre mice (Fig. 1a). Cre-dependent manipulations resulted in Rac1(CA) and EGFP specifically expression in PV or SST neurons of the brain. In these conditional knockin mice, we visualize Rac1(CA) through the fusion with EGFP. The EGFP fluorescence indicated the successful expression of Rac1(CA). Because many human diseases are resulted from heterozygous mutation, heterozygous PV-Rac1(CA)^−/+^ or SST-Rac1(CA)^−/+^ mice were then used in this study. Immunofluorescent staining of EGFP and PV or SST neurons confirmed that Rac1(CA) was expressed in PV and SST neurons, respectively (Fig. 1b). We sacrificed Rac1-loxp mice and examined the leaky EGFP neuron. We found there was no leaky expression, suggesting the labeling was specific (see supplementary Fig. 14).

Then, PV-Rac1(CA)^−/+^ and their control mice (PV-Cre and Rac1(CA)-loxp) were used in a three-chamber social behavioral test with 10 min per trial for three trials, in which the intertrial intervals (ITIs) between trial 2 and 3 were first set at 1 min. Neither sniff time (s) nor social index were different among the groups in trial 2 (see Supplementary Fig. 1a–c) and trial 3 (Fig. 1c–e), suggesting that Rac1(CA) in PV neurons does not affect social memory. Next, this behavioral test was then carried out with the ITIs at 5 min. We found that sociability in trial 2 was not different among the groups (see Supplementary Fig. 1d, e). However, in trial 3 after the ITIs 5 min, rapid forgetting was promoted in PV-Rac1(CA)^−/+^ mice as indicated by no significant difference in sniff time (s) on S1 and S2 mouse (Fig. 1f). And the social index of PV-Rac1(CA)^−/+^ mice is significantly lower (Fig. 1g). These results suggest that Rac1(CA) in PV neurons is sufficient for promoting rapid forgetting of social nmemory, leading to no preference on S2 over S1 mouse.

By contrast, Rac1(CA) in SST neurons had no effects on rapid forgetting of social memory as reflected by sniff time or social index without difference among the groups (Fig. 1h, i and see Supplementary Fig. 1f, g).

Interestingly, we also tested Rac1(CA) in PV neurons in a 2-trial object memory with the ITIs at 1 or 5 min between learning and test. We found that Rac1(CA) in PV but not SST neurons promoted rapid forgetting of object memory with ITIs at 5 min but not at 1 min (see Supplementary Fig. 2b–d), suggesting that this PV specific Rac1(CA) mechanism for rapid forgetting affects both social and object memory. However, neither open field nor elevated-plus maze test was affected by Rac1(CA) in PV or SST neurons relative to their control mice (see Supplementary Fig. 3). Thus, Rac1(CA) in PV but not SST neurons of the brain promoted rapid forgetting of both social and object recognition memory.

### Compared to the ACC and CA1, activating Rac1 in mPFC PV neurons facilitates rapid forgetting of social memory

Then we wanted to study the effects of Rac1 activity in PV or SST neurons on social related brain regions. We examined the c-Fos expression on the mPFC, ACC, and CA1 regions 1 h after the social behavior. The c-Fos expression in these brain regions was found higher in the behavioral tested than in naïve mice (see Supplementary Fig. 4), suggesting that these brain regions deserve further study.

**Fig. 2:**
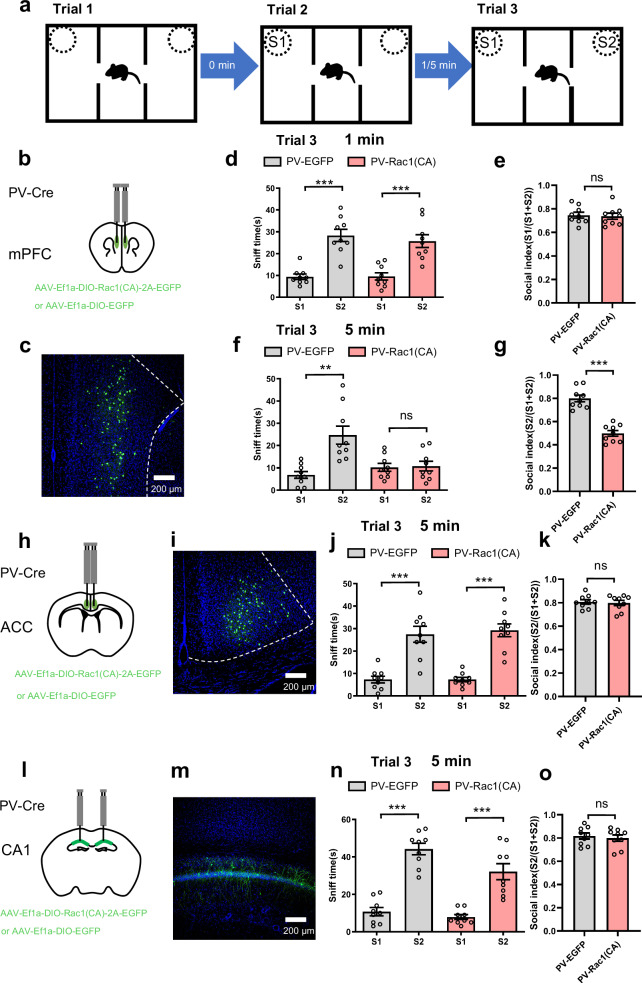
Compared to the ACC and CA1, activating Rac1 in mPFC PV neurons facilitates rapid forgetting of social memory. **a** Experimental design for social memory using three-chamber sociability and social memory test, for which we designed the intertrial intervals (ITIs) at either 1 or 5 min before trial 3. **b** Location of the virus infection in the mPFC. **c** Representative image of virus expression, expressing Rac1(CA) or EGFP in PV neurons of mPFC. **d**, **e** When ITI was set at 1 min, social preference for the S2 mouse as indicated by more sniff time to S2 than to the S1 mouse was significant both in control (PV-EGFP) and PV-Rac1(CA) mice (n = 9/per group; *F*_(3, 32)_ = 20.231; S1 vs S2, ***p < 0.001, one-way ANOVA) and similar social index among the groups (n = 9/per group, *p* = 0.797, unpaired t test, two tailed), suggesting that constitutive activation of Rac1 in PV neurons of mPFC had no effect on social memory. **f**, **g** When ITI was set at 5 min, social preference for the S2 mouse as indicated by more sniff time to S2 than to the S1 mouse was significant in control (PV-EGFP) but not in PV-Rac1(CA) mice (n = 9/per group; *F*_(3, 18)_ = 9.37, S1 vs S2, **p = 0.006, *p* = 0.397, Brown Forsythe ANOVA test). Social index was higher in control than in PV-Rac1(CA) mice (n = 9/per group; ***p < 0.001, unpaired t test, two tailed), suggesting that constitutive activation of Rac1 in PV neurons of mPFC caused rapid forgetting of social memory. **h** Location of the virus infection in ACC. **i** Representative image of virus expression, expressing Rac1(CA) or EGFP in PV neurons of ACC. **j**, **k** When ITI was set at 5 min, social preference for the S2 mouse as indicated by more sniff time to S2 than to the S1 mouse was significant both in control (PV-EGFP) and PV-Rac1(CA) mice (n = 9/per group; *F*_(3, 20)_ = 24.650; S1 vs S2, ***p < 0.001, Brown Forsythe ANOVA test) and similar social index among the groups (n = 9/per group, *p* = 0.754, unpaired t test, two tailed), suggesting that constitutive activation of Rac1 in PV neurons of ACC had no effect on social memory. **l** Location of the virus infection in hippocampal CA1 region. **m** Representative image of virus expression, expressing Rac1(CA) or EGFP in PV neurons of CA1. **n**, **o** When ITI was set at 5 min, social preference for the S2 mouse as indicated by more sniff time to S2 than to the S1 mouse was significant both in control (PV-EGFP) and PV-Rac1(n = 9/per group; *F*_(3, 21)_ = 35.78; S1 vs S2, ***p < 0.001, Brown Forsythe ANOVA test) and similar social index among the groups (n = 9/per group, *p* = 0.641, unpaired t test, two tailed), suggesting that constitutive activation of Rac1 in PV neurons of CA1 had no effect on social memory. All data showed as mean ± s.e.m.

Thus, we injected AAV-Ef1a-DIO-Rac1(CA)-2A-EGFP or AAV-Ef1a-DIO-EGFP bilaterally into the mPFC, ACC, and CA1 in PV-Cre mice. PV-Rac1(CA) and PV-EGFP in the mPFC exhibited similar social behaviors in trial 2 (see Supplementary Fig. 5a–d) and 3 (Fig. 2a–e) with the ITIs at 1 min. However, PV-Rac1(CA) and PV-EGFP in the mPFC showed similar sociability in trial 2 (see Supplementary Fig. 5e–g), but rapid forgetting was promoted in trial 3 with the ITIs at 5 min in PV-Rac1(CA) relative to PV-EGFP of the mPFC (Fig. 2f, g). Notably, other manipulations of PV-Rac1(CA) and PV-EGFP in the ACC or CA1 resulted no influences in trial 2 (see Supplementary Fig. 5h–m) and 3 (Fig. 2h–o), suggesting that rapid forgetting of social memory is not affected by Rac1(CA) in PV neurons of the ACC or CA1. However, object memory was found to be remained unaffected by PV-Rac1(CA) of the mPFC with the ITIs at 5 min (see Supplementary Fig. 6), suggesting that Rac1(CA) in PV neurons of the mPFC is unique for promoting rapid forgetting of only social memory.

We examined the c-Fos expression after Rac1(CA) expressed in mPFC, and found the c-Fos expression in mPFC increased, indicating the functions of PV neurons and E/I balance are changed (see supplementary Fig. 13).

**Fig. 3:**
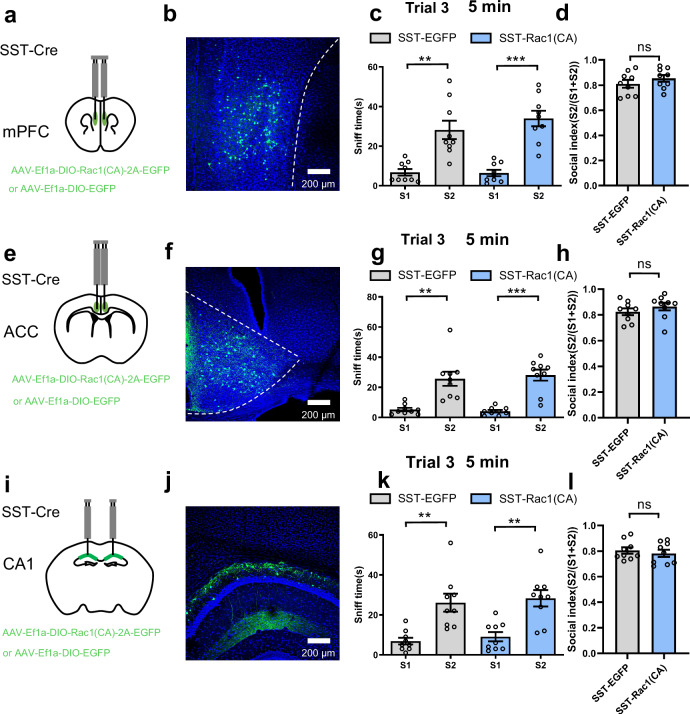
Activating Rac1 in mPFC SST neurons has no effect on rapid forgetting of social memory, similarly in ACC and CA1. **a** Location of the virus infection in mPFC. **b** Representative image of virus expression, expressing Rac1(CA) or EGFP in SST neurons of mPFC. **c**, **d** When ITI was set at 5 min, social preference for the S2 mouse as indicated by more sniff time to S2 than to the S1 mouse was significant both in control (SST-EGFP) and SST-Rac1 (n = 9/per group; S1 vs S2, **p = 0.003, ***p < 0.001, Kruskal-Wallis test) and similar social index among the groups (n = 9/per group, *p* = 0.301, unpaired t test, two tailed), suggesting that constitutive activation of Rac1 in SST neurons of mPFC had no effect on social memory. **e** Location of the virus infection in ACC. **f** Representative image of virus expression, expressing Rac1(CA) or EGFP in SST neurons of ACC. **g**, **h** When ITI was set at 5 min, social preference for the S2 mouse as indicated by more sniff time to S2 than to the S1 mouse was significant both in control (SST-EGFP) and SST-Rac1(n = 9/per group; *F*_(3, 17)_ = 17.550; S1 vs S2, **p = 0.006, ***p < 0.001, Brown Forsythe ANOVA test) and similar social index among the groups (n = 9/per group, *p* = 0.315, unpaired t test, two tailed), suggesting that constitutive activation of Rac1 in SST neurons of ACC had no effect on social memory. **i** Location of the virus infection in hippocampal CA1 region. **j** Representative image of virus expression, expressing Rac1(CA) or EGFP in SST neurons of CA1. **k**, **l** When ITI was set at 5 min, social preference for the S2 mouse as indicated by more sniff time to S2 than to the S1 mouse was significant both in control (SST-EGFP) and SST-Rac1 mice (n = 9/per group; *F*_(3, 22)_ = 11.030; S1 vs S2, **p_1_ = 0.007, **p_2_ = 0.002, Brown Forsythe ANOVA test) and similar social index among the groups (n = 9/per group, *p* = 0.552, unpaired t test, two tailed), suggesting that constitutive activation of Rac1 in SST neurons of CA1 had no effect on social memory. All data showed as mean ± s.e.m.

Furthermore, we injected AAV-Ef1a-DIO-Rac1(CA)-2A-EGFP or AAV-Ef1a-DIO-EGFP bilaterally into the mPFC, ACC, and CA1 in SST-Cre mice. No differences were found in the social behavioral tests between SST-Rac1(CA) and SST-EGFP mice in all of the brain regions (Fig. 3 and see Supplementary Fig. 7), suggesting that Rac1(CA) in SST neurons of these brain regions does not affect rapid forgetting of social memory.

For AAV-DIO-Ef1a-Rac1(CA)-2A-EGFP injection to PV-Cre mice, Rac1(CA) is fused with 2A peptide. The detection of 2A peptide indicated the expression of Rac1(CA). The immunofluorescence results showed that AAV-mediated Rac1(CA) expressed successfully in PV neurons of mPFC (see Supplementary Fig. 10).

We also injected AAV-Ef1a-DIO-Rac1(CA)-2A-EGFP or AAV-Ef1a-DIO-EGFP to CA2 of PV-Cre mice. No differences were found in social behavioral test between PV-Rac1(CA) and PV-EGFP group, indicating that activating Rac1 in PV neurons of social-related region CA2 also has no effects on forgetting of social memory (see supplementary Fig. 12).

### Inhibition of Rac1 in the mPFC PV neurons forms extended social memory

**Fig. 4:**
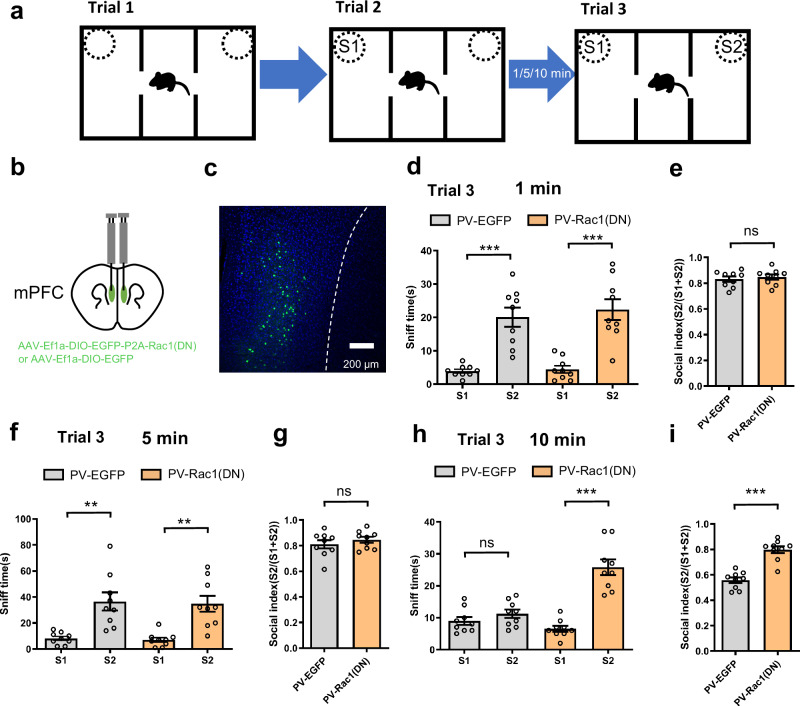
Inhibition of Rac1 in the mPFC PV neurons forms extended social memory. **a** Experimental design for social memory using three-chamber sociability and social memory test, for which we designed the inter-trial intervals (ITIs) at either 1, 5, or 10 min before trial 3. **b** Location of the virus infection in the mPFC. **c** Representative image of virus expression, expressing Rac1(DN) or EGFP in PV neurons of mPFC. **d**, **e** When ITI was set at 1 min, social preference for the S2 mouse as indicated by more sniff time to S2 than to the S1 mouse was significant both in control (PV-EGFP) and PV-Rac1(DN) mice (n = 9/per group; *F*_(3, 18)_ = 20.010; S1 vs S2, ***p < 0.001, Brown Forsythe ANOVA test) and similar social index among the groups (n = 9/per group, *p* = 0.574, unpaired t test, two tailed), suggesting that inhibition of Rac1 in PV neurons of mPFC had no effect on social memory. **f**, **g** When ITI was set at 5 min, social preference for the S2 mouse as indicated by more sniff time to S2 than to the S1 mouse was significant both in control (PV-EGFP) and PV-Rac1(DN) mice (n = 9/per group; *F*_(3, 18)_ = 11.600; S1 vs S2, **p_1_ = 0.006, **p_2_ = 0.004, Brown Forsythe ANOVA test) and similar social index among the groups (n = 9/per group, *p* = 0.396, unpaired t test, two tailed), suggesting that inhibition of Rac1 in PV neurons of mPFC had no effect on social memory. **h**, **i** When ITI was set at 10 min, social preference for the S2 mouse as indicated by more sniff time to S2 than to the S1 mouse was significant in PV-Rac1(DN) but not in control (PV-EGFP) mice (n = 9/per group; *F*_(3, 20)_ = 29.150; S1 vs S2, ***p < 0.001, *p* = 0.547, Brown Forsythe ANOVA test). Social index was higher in PV-Rac1(DN) than in control mice (n = 9/per group; ***p < 0.001, unpaired t test, two tailed), suggesting that inhibition of Rac1 in PV neurons of mPFC retarded rapid forgetting of social memory. All data showed as mean ± s.e.m.

The above results suggested that Rac1(CA) in PV neurons of the mPFC was unique and sufficient for promoting rapid forgetting of social memory. To address whether Rac1 activity in PV neurons of the mPFC was also necessary for rapid forgetting of social memory, we injected AAV-Ef1a-DIO-Rac1(DN)-p2A-EGFP or AAV-Ef1a-DIO-EGFP into the mPFC of PV-Cre mice. We found that PV-Rac1(DN) of the mPFC prevented rapid forgetting of social memory even though the ITIs between trial 2 and 3 were set at 1 (Fig. 4a–e and see Supplementary Fig. 8a–d), 5 (Fig. 4f, g and see Supplementary Fig. 8e, f), and 10 min (Fig. 4h, i and see Supplementary Fig. 8g, h). Notably, PV-EGFP as control mice showed rapid forgetting of social memory with the ITIs at 10 min (Fig. 4h, i and see Supplementary Fig. 8g, h). These results suggest that Rac1 activity is also necessary for rapid forgetting of social memory.

### PV but not SST neurons of the mPFC generated dual calcium peaks to delineate each social interaction event

The c-Fos study showed that more PV neurons were activated than SST neurons 1 h after social memory formation (see supplementary Fig. 11), indicating PV neurons plays a more important role in social memory.

**Fig. 5:**
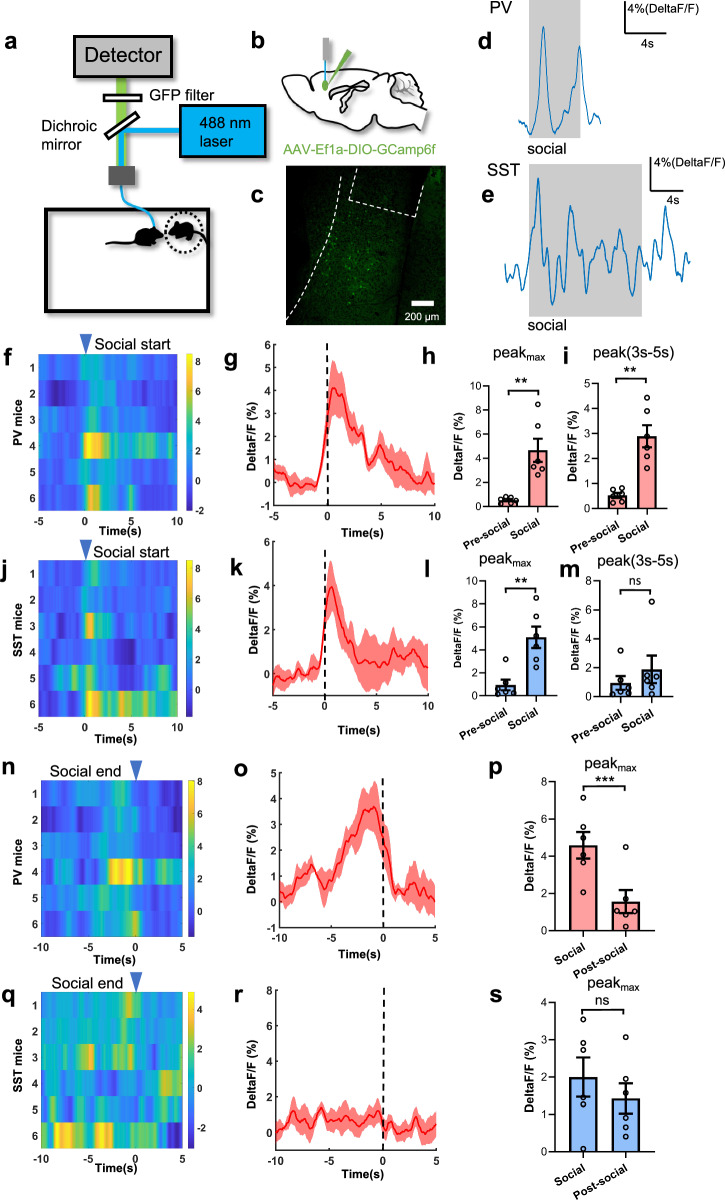
Different calcium activities of the mPFC PV and SST neurons in social behavior. **a** Schematic diagram of the photometry set up to record calcium activities of PV and SST neurons in social behavior. **b** Location of the virus infection. **c** Representative image of the virus expression in mPFC. **d**, **e** Representative Ca^2+^ signals of PV or SST neurons in social episodes (grey area). **f**–**h** PV neurons in mPFC were activated at the onset of the social (blue triangle mark), indicated by comparison of PV neuron maximum peaks between pre-social and social period (n = 6/per group, **p = 0.008, paired t-test, two tailed). Each row of the heatmap represents one mouse. The color scale at the right indicate Delta F/F(%). Thick lines indicate mean and shaded area indicate ± s.e.m. **i** Ca^2+^ activity of PV neurons was higher in the 3–5 s period of social than pre-social period, indicated by comparison of PV neuron peaks between pre-social period and 3–5 s period of social (n = 6/per group, **p = 0.003, Wilcoxon test, two tailed). **j**–**l** SST neurons in mPFC were activated at the onset of the social (blue triangle mark), indicated by comparison of SST neuron peaks between pre-social and social period (n = 6/per group, **p = 0.008, paired t-test, two tailed). Each row of the heatmap represents one mouse. The color scale at the right indicate Delta F/F(%). Thick lines indicate mean and shaded area indicate ± s.e.m. **m** Ca^2+^ activity of SST neurons was comparable between the 3–5 s period of social and pre-social period, indicated by comparison of SST neuron peaks between pre-social period and 3–5 s period of social (n = 6/per group, *p* = 0.438, Wilcoxon test, two tailed). **n**–**p** Peaks of Ca^2+^ signals in PV neurons arise at the end of social behavior (blue triangle mark), indicated by comparison of PV neuron maximum peaks between social and post-social period (n = 6/per group, ***p < 0.001, paired t-test, two tailed). Each row of the heatmap represents one mouse. The color scale at the right indicate Delta F/F(%). Thick lines indicate mean and shaded area indicate ± s.e.m. **q**–**s** Ca^2+^ signals of SST neurons was comparable at the end of social behavior (blue triangle mark), indicated by comparison of SST neuron maximum peaks between pre-social and social period (n = 6/per group, *p* = 0.482, paired t-test, two tailed). Each row of the heatmap represents one mouse. The color scale at the right indicate Delta F/F(%). Thick lines indicate mean and shaded area indicate ± s.e.m. All data showed as mean ± s.e.m.

To detect the different real-time activities of PV and SST neurons in social behavior, we used fiber photometry to record calcium signals of PV or SST neurons of the mPFC in the social period, following the mPFC of PV-Cre or SST-Cre mice transinfected by AAV-Ef1a-DIO-GCamp6f (Fig. 5a–e). At the same time, all behaviors during social were recorded. Calcium signals and social behavioral events were overlaid together (see Supplementary Fig. 9). Thus, calcium signals during the onset and the end of each social interaction event during 10 min were aligned and quantitively analyzed. We found that calcium signals from PV neurons showed dual peaks at the start (Fig. 5f–i) and at the end (Fig. 5n–p) of each social interaction event, but SST neurons showed only one peak at the start of the event (Fig. 5j–m, q–s). This finding suggests that PV neurons’ activation delineates each social interaction event, implicating that PV neurons may generate in a precise timing manner linked with social behavior.

We calculated the AUC of calcium signals and found that the PV neurons had higher AUC in social period, compared to pre-social or post-social period (see supplementary Fig. 15a, b). However, there was no significant difference in SST neurons between social and pre-social period, similarly in social and post-social period (see supplementary Fig. 15c, d). These results suggested that PV neurons played a more important role in social behavior than SST neurons.

Previous report suggests that Rac1 activity can trigger intracellular calcium release [24]. Thus, we speculated that Rac1(CA) or Rac1(DN) in PV neurons of the mPFC may stimulate or extinguish the dual timing of calcium signals with social behavior, eventually for promoting or preventing rapid forgetting of social memory.

## Discussion

In the present study, we identified for the first time that PV-specific Rac1 activity of the mPFC was both necessary and sufficient for rapid forgetting of social memory. This is likely attributable to activities of PV neurons of the mPFC that display dual calcium peaks linked with each social interaction event. Since Rac1 activity in the PV neurons may regulate their neuronal activity patterns, a precise timing of PV neurons with a social event may endow their capability for encoding this event. Thus, we have speculated that these PV neurons may be the important components of the engram for social memory but altered activities of these PV neurons by Rac1(CA) or Rac1(DN) may lead to the promotion or prevention of rapid forgetting of social memory.

### PV neurons would be chosen for processing social memory

PV neurons often fire at high frequency (e.g. > 30 Hz) trains of action potentials (APs) in a theta phase-locked-in manner [18, 25–27]. In this study, using fiber photometry we found that PV neurons of the mPFC displayed dual peaks of calcium signals at the start and the end of each social interaction event that lasts for only several seconds, suggesting that there was a precise timing of PV-neuron firing with the social event. Thus, we speculated that this precise timing would have caused short-term plasticity for social memory. In contrast, even if other neurons such SST or glutamatergic neurons could be also important for this process, it remains still very unlikely because these neurons usually fire at low frequency (e.g. 2 Hz) [18, 28], providing insufficient time resolution for dealing such a short event. Therefore, we believe that this is the reason why PV neurons are chosen for processing social memory.

PV and SST neurons are two major subtypes of GABAergic neurons. Previous studies showed that other GABAergic neurons, such as VIP neurons, which has a low propotion compared to PV and SST neurons [16, 29]. So we choose PV and SST neurons in our study.

### Rapid forgetting depended on both the ITIs and Rac1 activity

We generated mouse lines by knocked-in Rac1(CA) to PV or SST neurons in the brain or in the brain regions including the mPFC, ACC, and CA1. Only PV-neurons specific Rac1(CA) in the brain or the mPFC promoted rapid forgetting of social memory when the intervals were set at 5 min but not at 1 min, suggesting that this type of rapid forgetting did not occur until the interval was prolonged to 5 min. The controls such as Cre or loxp mice were used here to demonstrate that no rapid forgetting occurred under these conditions. Notably, the controls did not occur rapid forgetting until the interval was prolonged to 10 min (see Fig. 5h, i and Supplementary Fig. 8g, h), which is highly consistent with our previous report.

Therefore, rapid forgetting of social memory did not usually occur in a short interval (1–5 min) but occurred in a longer interval (10 min) between learning and test. However, we found that Rac1(CA) in PV neurons of the brain or mPFC enabled rapid forgetting of social memory with the interval at 5 min, suggesting that this CA variant of Rac1 promoted rapid forgetting. It is unclear whether our manipulations of Rac1(CA) influenced the endogenous wildtype Rac1 activity or not, though this needs more studies in the future. We here only focused on the concept that PV neurons-specific Rac1(CA) of the mPFC promoted rapid forgetting because they were very likely involved in the processing of social memory with a unique time resolution. Rac1(CA) would have certain influences on the activities of PV neurons and disrupt time resolution or precise timing.

Notably, much stronger evidence came from the manipulation of Rac1(DN) specifically knocked in PV neurons of the mPFC. This variant of Rac1 led to no rapid forgetting of social memory even though the controls exhibited rapid forgetting of the memory with the interval at 10 min, suggesting that Rac1 activity might be necessary for rapid forgetting of social memory. Taken together, we conclude for the first time that Rac1 activity in PV neurons of the mPFC is both necessary and sufficient for rapid forgetting of social memory.

The duration of social memory depends on the length of the social interaction period, when the test mice get familiar to stranger 1. In a previous study, after a 2 h familiarization session, the rapid forgetting of social memory occurs in the first 30 min [30], indicated by the test mice couldn’t discriminate the familiar and novel mice after 30 min. In our study, the familiarization session only lasted for 10 min, resulting in the duration of social memory being shorter than in the previous studies.

After the learning process, the memory is in a fragile state. When we test the memory soon after learning (e.g., a 1-min interval), animal may rely on the short-term or working memory. When the interval is extended to 5 or 10 min, the memory begins to consolidate from short-term memory to the early stage of long-term memory. When the social memory can’t be consolidated well, the rapid forgetting of social memory occurs.

Then how does the Rac1 activity integrate rapid forgetting process within minutes. Rac1 is a member of Rho-GTPase, which act as a molecular switch involving in forgetting of memory. When Rac1 is activated, it can trigger the downstream PAK and LIMK signal pathway quickly, leading to the changes in the size and shape of the dendrite spines. A previous study showed that Rac1 and other Rho GTPase responds to synaptic stimulation within seconds [31], acting the downstream pathway that reorganize actin in dendrite spines within minutes, which is essential to synaptic plasticity. In this study, the transient volume change was observed in spines, which may contribute to rapid forgetting within minutes.

The similar way comes for physiological activities of Rac1, according to the previous study [31], Rac1 can be activated within seconds and trigger the downstream pathway within minutes. As for the rapid forgetting process of social memory, innate Rac1 may be activate and trigger the downstream PAK and LIMK signal pathway within minutes, leading to transient spine enlargement.

### Rapid forgetting of social memory depended on both PV neuron and the mPFC

It was surprising to us that Rac1(CA) in PV neurons of the ACC or CA1 did not affect rapid forgetting of social memory, as these brain regions have been all known to be critical for social memory [6, 9, 13] but whether they are critical for its rapid forgetting remained unclear. Here, we tried to provide an explanation, for which rapid forgetting might have been involved in certain brain regions, especially the mPFC and possibly its neural circuits. It is well-known that the mPFC is critical for many cognitive functions such as working memory, emotion, social memory, social dominance [32], and etc. Notably, the mPFC-amygdala or the mPFC-nucleus accumbens pathway are identified to be critical for regulating social behavior [33, 34]. Thus, we believed that PV neurons specific Rac1(CA) of the mPFC may also influence the neural circuits of the mPFC that are actually responsible for rapid forgetting of social memory.

Rapid forgetting of other types of memories may involve distinct brain regions and their neural circuits. This assumption is based on the finding that PV-neurons specific Rac1(CA) in the brain or in the mPFC promoted rapid forgetting of both social and object memory or that of only social memory, respectively. The brain region responsible for object recognition may be other brain regions, resulting activating Rac1 in PV neurons has no effects on rapid forgetting of object memory. These results indicate the specific functions of brain regions. In other words, other brain regions rather than the mPFC could be critical for object memory and its rapid forgetting.

For other social related regions such as CA2, We found that Rac1(CA) in PV neurons of CA2 had no effects on rapid forgetting of social memory (see supplementary Fig. 12). As for ventral CA1, A previous study showed that ventral CA1 participated in long-term social memory [30]. It seems ventral CA1 is possibly relate to the forgetting of long-term social memory.

Therefore, we thought that rapid forgetting of social memory might have required Rac1 activity only in PV neurons of the mPFC. Other possibilities cannot be ruled out. Either ACC or CA1 has been suggested to be critical for retrieval of long-term memories. For example, Rac1 activity in glutamatergic neurons of the CA1 promotes the forgetting of long-term but not short-term social memory [23]. Another possibility is that these brain areas may influence other aspects of cognitive functions to lead to ASD-like social dysfunctions [35].

To understanding the role of PV neurons in social memory and forgetting, we examined c-Fos positive neurons in PV or SST neurons after social memory formation. We found that the number of c-Fos positive neurons were significantly more in PV neurons than SST neurons. These results indicate that PV but SST neurons may act as engram cells in the social memory and control rapid forgetting of social memory. When we activate Rac1 in PV neurons, the engram cell and the entire network were changed, resulting in the forgetting of social memory (see supplementary Fig. 11).

To understand how Rac1(CA) affect the the activity of PV neuron. We examined the c-Fos expression after Rac1(CA) expressed in mPFC, and found the c-Fos expression in mPFC increased, indicating the functions of PV neurons and E/I balance were changed (see supplementary Fig. 13). These results indicated that Rac1(CA) affect the PV neuron related neural network to facilitate rapid forgetting of social memory.

In summary, this study strongly suggests that Rac1 activity in PV neurons of the mPFC is responsible for rapid forgetting of certain social memories. Notably, more (e.g. CA) or less (e.g. DN) Rac1 activity in these neurons is potentially as the therapeutic target to promote or prevent this type of forgetting, which is often associated with social dysfunctions in many brain disorders.

## Materials and methods

### Mouse husbandry and handling

Male mice above 9 weeks were used for behavior test and mice above 6 weeks were used for virus injection. Mice were housed on a 12 h light cycle. The feeding conditions were as follows: a 12/12-h light/dark cycle, a thermoregulated environment; free access to water and food. Mice carrying either PV-Cre (017320, JAX, PV-Cre) or SST-Cre (018973, JAX, SST-Cre) allele were crossed with Rac1-loxp mice (012361, JAX, Rac1-loxp), resulting in male and female F1 hybrid offspring. All the transgenic mice we used have a C57BL/6J genetic background. After weaning, all mice were group-housed (3–5 mice per cage). All husbandry and experiment procedures were reviewed and approved by the animal ethics committee of Kunming Institute of Zoology, Chinese Academy of Sciences (No. SMKX-20160301-01).

In Rac1-loxp mice, the conditional allele was targeted to Gt(ROSA)26Sor locus and has a loxP-flanked Neo-STOP cassette preventing transcription of the downstream Rac1(G12V) and EGFP sequence. When Rac1-loxp mice was crossed with PV-Cre or SST-Cre mice, the Cre recombinase deleted the Neo-STOP cassette and lead to the expression of Rac1(G12V) and EGFP.

### Open Field Test

Mice were habituated for 20 min in the test room before the trial began. Then the mice were put in an open field arena (29 cm long, 29 cm wide, and 20 cm high) and explored for 1 h. The total distance traveled was recorded and analyzed by the med system to measure locomotor activity.

### Elevated Plus Maze

Mice were habituated for 20 min in the test room before the trial began. The elevated plus maze is a cross-shaped platform with two open and two closed arms placed 75 cm above the ground. During the trial, the mouse was placed in the middle of the platform with its head toward the open arm. Then the mice was allowed to freely explore the new environment for 10 min. The activity of the mice was recorded and the time spent in the open arms or the closed arms was measured.

### Three chamber social recognition test

The apparatus consisted of a three-compartment (Length: 40 cm; Width: 20 cm; Height: 24 cm for each) white Plexiglas box, with openings (7 cm wide) allowing access into each chamber. The animals were allowed to acclimate to the test room for at least 20 min before the trial began. Two identical wire cups (diameter 10 cm) were placed in the corner of each side compartment (one per site) during testing sessions. In the first trial, a test mouse was allowed to explore the empty cups and the entire apparatus for 10 min. After habituation, the mouse was confined to the center chamber. In the second trial, a stranger 1 younger than the test mouse (to avoid aggressive behavior) was placed inside the wire cup in one of the compartments. The test mouse was allowed to freely explore all three compartments of the apparatus for another 10 min. Then the mouse was confined to the center chamber for an inter-trial interval (ITI) of 1 min, 5 min, or 10 min and a stranger 2 was placed in the other wire cup. In the third trial, both doors to the side chambers were reopened and the subject was allowed to explore the entire apparatus for 10 min. The behavior of the mouse were recorded by the Corel VideoStudio and the time sniffing the stranger or the wire cup was recorded. The social index were calculated as the time sniffing stranger 2 divided by the total time sniffing stranger 2 and stranger 1.

### Object recognition

Animals were allowed to acclimate to the behavioral testing room for at least 20 min before the trial began. The object recognition box was a rectangular apparatus (60 cm long × 40 cm wide × 24 cm high) made from white Plexiglas. Two identical objects (X) were placed in opposite corners at approximately 5 cm distance from the wall. Each mouse was placed individually in the third corner, facing the wall, and was allowed to explore for 10 min. After an inter-trial interval (ITI) of 5 min, the mouse was reintroduced to the box for trial 2 with one identical object (X) and one different object(Y). Again the mouse was allowed to explore for 10 min. The behavior of the mouse were recorded by the Corel VideoStudio and the time spent with the object was recorded. The discrimination index was calculated using the formula (Y − X)/(X + Y).

### Virus injection

These viruses we used contain a DIO vector, where the transgene is floxed by two pairs of recombination sites. When Cre is introduced to the virus, it binds to one pair of lox sites, and flips the Rac1(CA)-EGFP, Rac1(DN)-EGFP or EGFP into the correct orientation for transcription. Mice were anesthetized using pentobarbital sodium (1% concentration) and standard stereotactic procedures were used. Viruses were injected using a pulled-glass capillary at a slow rate of 100 nl min^−1^ (Nanoliter 2010, WPI; Micro4 controller, WPI). The glass capillary was withdrawn 10 min after the cessation of injection. 350 nl of AAV-Ef1a-DIO-Rac1(CA)-EGFP or AAV-Ef1a-DIO-EGFP (OBiO technology) was bilaterally injected into the mPFC (distance from bregma, AP 2.0 mm, ML ± 0.30 mm; distance from cortex, DV − 2.0 mm) of PV-Cre or SST-Cre mice and incubated for 2 weeks before downstream experiments. 500 nL of AAV-Ef1a-DIO-Rac1(CA)-2A-EGFP or AAV-Ef1a-DIO-EGFP (OBiO technology) was bilaterally injected into the ACC of PV-Cre or SST-Cre mice (distance from bregma, AP 0.62 mm, ML ± 0.25 mm; distance from cortex, DV − 1.5 mm) and incubated for 2 weeks before behavioral experiments. 500 nL of AAV-Ef1a-DIO-Rac1(CA)-EGFP or AAV-Ef1a-DIO-EGFP (OBiO technology) was bilaterally injected into the CA1 of PV-Cre or SST-Cre mice (distance from bregma, AP − 2.0 mm, ML ± 1.5 mm; distance from cortex, DV − 1.3 mm) and incubated for 2 weeks before behavioral experiments. 350 nl of AAV-Ef1a-DIO- EGFP-P2A-Rac1(DN) or AAV-Ef1a-DIO-EGFP (OBiO technology) was bilaterally injected into the mPFC (distance from bregma, AP 2.0 mm, ML ± 0.30 mm; distance from cortex, DV − 2.0 mm) of PV-Cre mice and incubated for 2 weeks before downstream experiments. The virus titer of AAV-Ef1a-DIO-Rac1(CA)-EGFP was 5.24 + E12 vg/mL. The virus titer of AAV-Ef1a-DIO-EGFP was 4.29 + E12 vg/mL. The virus titer of AAV-Ef1a-DIO- EGFP-P2A-Rac1(DN) was 3.33 + E12 vg/mL. Rac1(CA) refers to Rac1(G12V) and Rac1(DN) refers to Rac1(T17N).

### Fiber implantation and fiber photometry recording

After AAV-Ef1a-DIO-GCamp6f injection, Mono optic fiber (125 mm O.D., 0.37 numerical aperture (NA); Newdoon Inc.) was implanted unilaterally 200 μm above the virus injection site of mPFC in PV-Cre mice and SST-Cre mice (AP 2.0 mm, ML ± 0.30 mm; distance from cortex, DV − 1.8 mm). Once positioned above the virus inject position, the mono optic fiber was cemented using dental cement to the skull, which contained three screws that lay medially to the implant site. Mice were housed for 2 weeks for recovery. Mice were handled by the investigator 2–3 d before behavioral experiments. Fiber photometry system (ThinkerTech, Nanjing) was used to record calcium signals from genetically identified neurons including PV and SST neurons. An optical fiber (125 mm O.D., NA = 0.37, 1 m long) guided the 488 nm light to implanted optical fiber.

The laser power was adjusted at the tip of optical fiber to a low level of 0.01–0.02 mW, to minimize bleaching. Behaviors were recorded by a camera and social epochs from the recorded mice were manually annotated. Ca^2+^ signal was aligned to the onset of social behavior. For social beginning period, 0 was the starting point of social behavior, which was the time when the mice started sniffing the stranger. The 5 s before and 10 s after social behavior were selected for statistics. Baseline activities were calculated from −5–−3 s before each social epoch. The social activities were calculated from 0–5 s after the social starting point. The calcium signals were indicated by DeltaF/F. The peak or average AUC of fluorescence activity of PV or SST neurons between the baseline and the stages after social interaction was compared. For the end of social period, 0 was the endpoint of social behavior when the mice stopped social. The 10 s before and 5 s after the endpoint were selected for statistics. The social activities were calculated from −5–0 s before the social endpoint. The post-social activities were calculated from 1–3 s after the social endpoint. The calcium signals was indicated by DeltaF/F. The peak or average AUC of fluorescence activity of PV or SST neurons between the social and the post-social period was compared. Data analysis was performed using MATLAB (MathWorks).

### Immunohistochemistry

Mice were transcardially perfused with 20 mL phosphate-buffered saline (PBS), followed by 20 mL 4% paraformaldehyde (pH 7.4). Dissected brains were post-fixed in 4% paraformaldehyde at 4 °C and then placed in a 30% sucrose solution at 4 °C until the brains sank. Using a cryostat (Leica CM1950), 50 μm-thick coronal sections were collected in PBS. Sections were washed in PBS three times (10 min each time) and then incubated with blocking solution (5% BSA, and 0.15% Triton X-100 in PBS) for 1 h at room temperature. Sections were then incubated with primary antibody in blocking solution overnight at 4 °C. The following primary antibodies were used: mouse anti-parvalbumin (1:500; sigma, SAB4200545), rat anti-somatostatin (1:400; Millipore, MAB354), rabbit anti-EGFP (1:500; Invitrogen, 20077). After washing in PBS three times (10 min each time), sections were then incubated with species-specific fluorophore-conjugated secondary antibodies (1:1000, goat anti-mouse Alexa Fluor 594, Invitrogen, ab150080; 1:1000, goat anti-Rabbit Alexa Fluor 488, ab150077; 1:1000, goat anti-Rat Alexa Fluor 647, A-21247) in blocking solution for 2 h at room temperature. After washing in PBS three times (10 min each time), sections were mounted on glass slides. Using a confocal microscope (Olympus FV1200) with a 40X objective and zoom 2 in 1024 × 1024 pixels, images were acquired to verify Rac1(CA) expression. For the detection of social related brain areas using c-Fos immunostaining, mice were perfused 1 h later after 1 h home-cage social interaction. Same immunostaining procedures were performed and c-Fos antibody (1:1000; CST, 2250S) was used. To excluded other factors, mice were housed one mouse/per cage. 4 sections were collected per mouse and images were acquired using a confocal microscope (Olympus, FV1200) at a 20X objective. Cell counts were performed using ImageJ software. For other c-Fos immunostaining experiments, c-Fos antibody (1:1000; SYS-226-017) were used and mice were group housed (3–5 mice/per cage).

### Quantification and statistical analysis

Animals were randomly assigned to groups by permuting their identification numbers. The investigator was not blinded to the group allocation during the animal experiment. When assessing the outcome, the investigator was blind. Mice with abnormal social behavior in trial 2 of the social recognition test were excluded. The animal behavior experiments were repeated with three different batches of animals.

All data were shown as mean ± SEM and the statistical analyses were done with SPSS or Prism 7 (GraphPad). For 2 groups, normality of the data was tested using the Shapiro-Wilk normality test. Data with normal distribution were analyzed by two tailed unpaired t-test or paired t-test. Nonparametric unpaired data were compared using Mann-Whitney test, and nonparametric paired data were compared using the Wilcoxon test. For 3 groups or more, normality of the data was tested using the Shapiro-Wilk normality test. The variance of the data with normal distribution was calculated. If the variance is equal, ordinary one way ANOVA test was made with Bonfferroni for multiple comparisons. If the variance is not equal, the Brown Forsythe ANOVA test was made with Dunnett’s correction for multiple comparisons. Nonparametric data were analyzed by the Kruskal-Wallis test with Dunnett’s correction for multiple comparisons. p < 0.05 was considered statistically significant. *p < 0.05, **p < 0.01, ***p < 0.001.

## Supplementary information

Supplementary Figures

## Data Availability

All raw data sets can be made available upon reasonable request.
