# Supplementary material for: Rac1 in parvalbumin neurons of the medial prefrontal cortex governs rapid forgetting of social memory
Source: Mol Psychiatry. 2025 Mar 29;30(8):3740–51. doi: 10.1038/s41380-025-02963-9 (PMC12240863; doi:10.1038/s41380-025-02963-9)
Supplement: Supplementary file 1 — Supplementary Figures [file 41380_2025_2963_MOESM1_ESM.pdf]

Supplementary Fig. 1

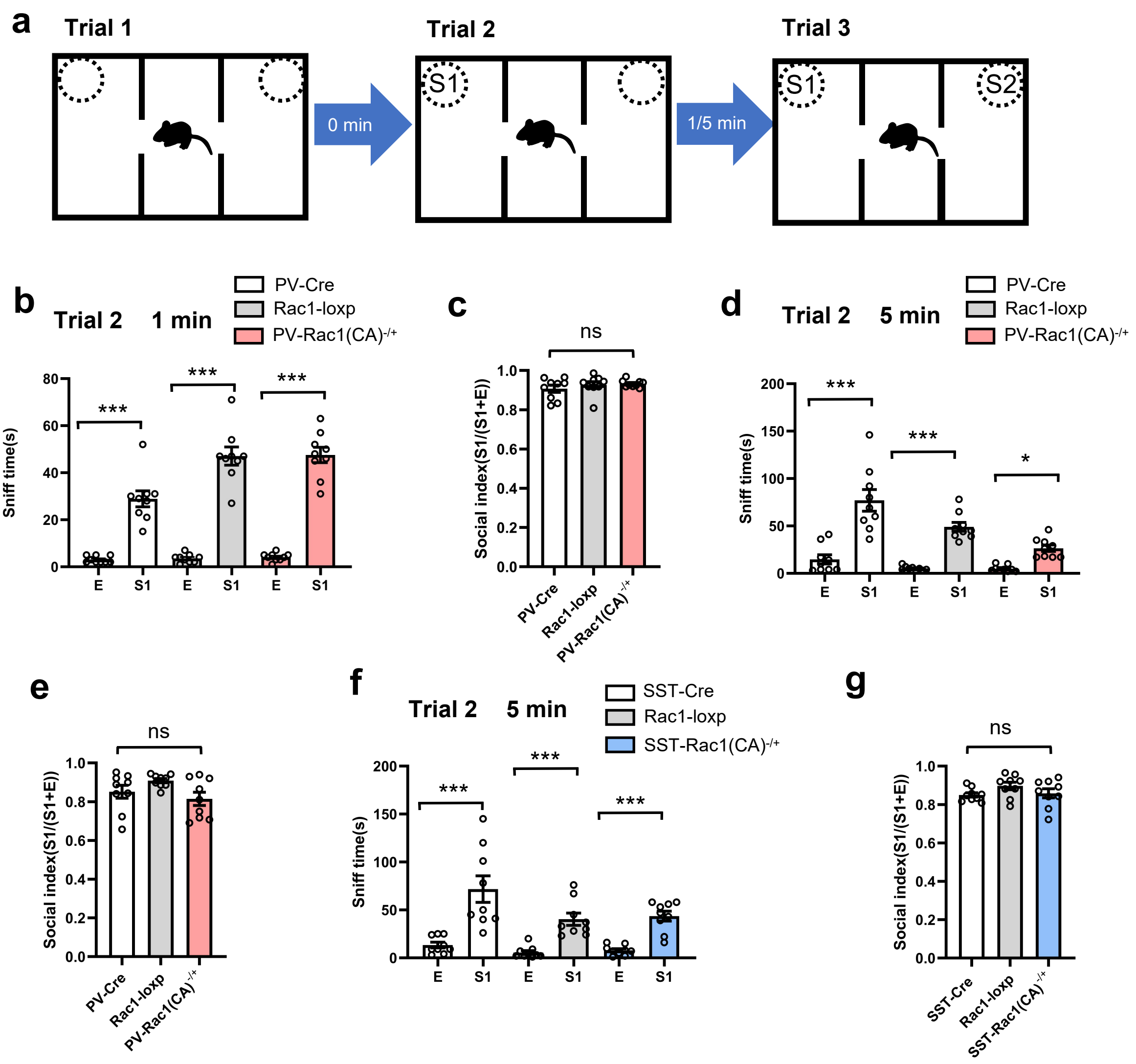

Supplementary Fig. 1: Rac1(CA) in PV or SST neurons has no effect on sociability. (a) Experimental design for social memory using three-chamber sociability and social memory test, for which we designed the inter-trial intervals (ITI) at either 1 or 5 min before trial 3. (b-c) When ITI was set at 1 min, PV-Cre, Rac1-loxp, and PV-Rac1(CA)$^{-/+}$ mice showed comparable sociability to stranger 1 (S1) mouse in trial 2 as indicated by more sniff time to S1 mouse than to empty wire cup (E) (n = 9/per group; S1 vs E, ***$p < 0.001$, Kruskal-Wallis test) and similar social index among the groups (n = 9/per group, $p = 0.681$. Kruskal-Wallis test), suggesting that constitutive activation of Rac1 in PV neurons had no effect on sociability. (d-e) When ITI was set at 5 min, PV-Cre, Rac1-loxp, and PV-Rac1(CA)$^{-/+}$ mice showed comparable sociability to stranger 1 (S1) mouse in trial 2 as indicated by more sniff time to S1 mouse than to empty wire cup (E) (n = 9/per group; S1 vs E, ***$p < 0.001$, *$p = 0.018$, Kruskal-Wallis test) and similar social index among the groups (n = 9/per group, $F_{(2, 24)} = 2.8$, p=0.0787. one-way ANOVA), suggesting that constitutive activation of Rac1 in PV neurons had no effect on sociability. (f-g) When ITI was set at 5 min, SST-Cre, Rac1-loxp, and SST-Rac1(CA)$^{-/+}$ mice showed comparable sociability to stranger 1 (S1) mouse in trial 2 as indicated by more sniff time to S1 mouse than to empty wire cup (E) (n = 9/per group; S1 vs E, ***$p < 0.001$, Kruskal-Wallis test) and similar social index among the groups (n = 9/per group, $F_{(2, 24)} = 1.732$, $p = 0.1983$. one-way ANOVA), suggesting that constitutive activation of Rac1 in SST neurons had no effect on sociability.

Supplementary Fig. 2

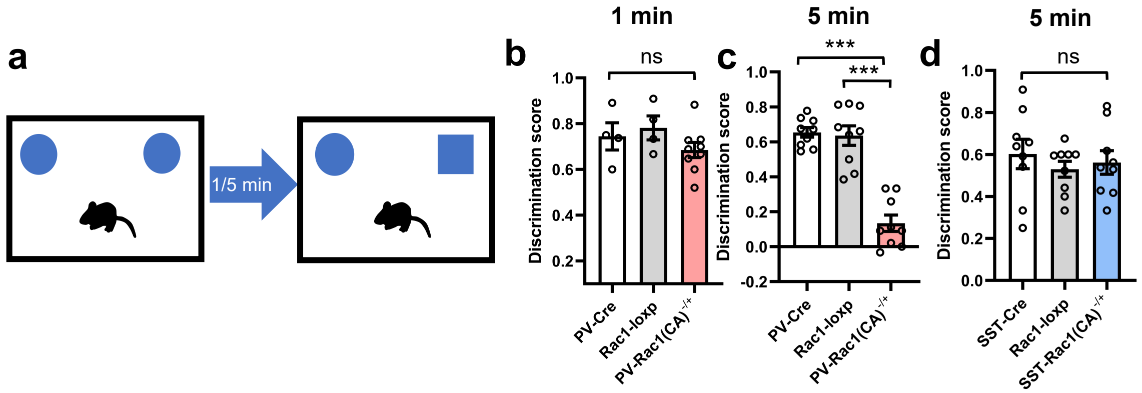

Supplementary Fig. 2: Rac1(CA) in PV but not SST neurons promotes rapid forgetting of object memory. (a) Experimental design for object recognition memory with the inter-trial intervals (ITIs) at 1 or 5 min between learning and test trial. (b) When ITI was set at 1 min, object memory of control (PV-Cre or Rac1-loxp) and PV-Rac1(CA)$^{-/+}$ mice was comparable as indicated by that object memory was comparable in both control (PV-Cre or Rac1-loxp) and PV-Rac1(CA)$^{-/+}$ mice (PV-Cre and Rac1-loxp, n = 4, PV-Rac1(CA)$^{-/+}$, n=9, $F_{(2, 24)} = 1.304$, $p = 0.3026$, one-way ANOVA). (c) When ITI was set at 5 min, rapid forgetting of object memory in PV-Rac1(CA)$^{-/+}$ mice occured as indicated by that object memory of PV-Rac1(CA)$^{-/+}$ mice showed deficits in 5 min interval object recognition(n=9, $F_{(2, 24)} = 42.84$, $***p < 0.001$, one-way ANOVA). (d) When ITI was set at 5 min, rapid forgetting of object memory in SST-Rac1(CA)$^{-/+}$ mice didn't occur as indicated by that object memory was comparable in control (SST-Cre or Rac1-loxp) and SST-Rac1(CA)$^{-/+}$ mice (n=9/group, $F_{(2, 24)} = 0.4224$, $p = 0.6603$, one-way ANOVA).

Supplementary Fig. 3

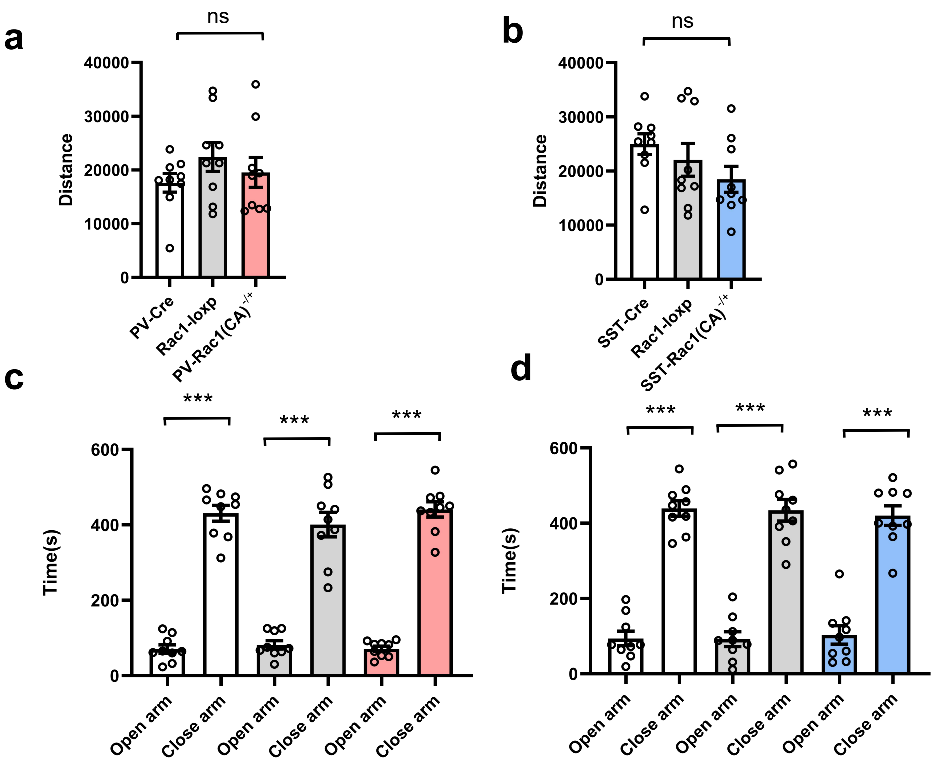

Supplementary Fig. 3: Rac1(CA) in PV or SST neurons has no effect on open field and elevated-plus maze test. (a) Comparable locomotion as indicated by comparable distance traveled by PV-Rac1(CA)$^{-/+}$ and control mice (PV-Cre or Rac1-loxp) (n=9, p = 0.4058, Kruskal-Wallis test). Bars represent mean ± s.e.m. (b) Comparable locomotion as indicated by comparable distance traveled by SST-Rac1(CA)$^{-/+}$ and control mice (PV-Cre or Rac1-loxp) (n=9, $F_{(2, 24)}$ =1.701, p = 0.2038, one-way ANOVA). Bars represent mean ± s.e.m. (c) Comparable anxiety level as indicated by comparable time spent by PV-Rac1(CA)$^{-/+}$ or control mice (PV-Cre or Rac1-loxp) in open and close arm (n=9, $F_{(5, 26)}$ =100.1, ***p < 0.001, Brown Forsythe ANOVA). Bars represent mean ± s.e.m. (d) Comparable anxiety level as indicated by comparable time spent by SST-Rac1(CA)$^{-/+}$ or control mice (PV-Cre or Rac1-loxp) in open and close arm (n=9, $F_{(5, 48)}$ =61.36, ***p < 0.001, one-way ANOVA). Bars represent mean ± s.e.m.

Supplementary Fig. 4

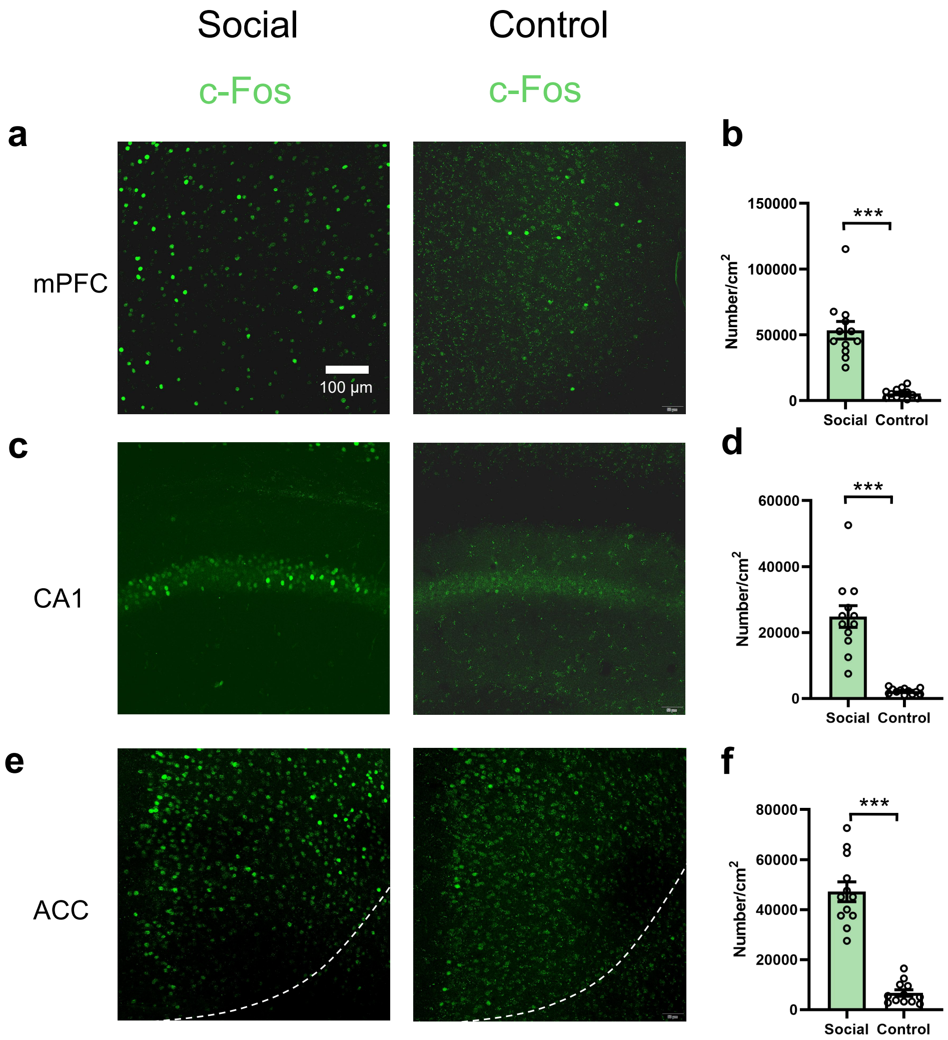

Supplementary Fig. 4: Neurons in mPFC , ACC and CA1 are activated in social behavior. (a) Representative images of c-Fos-positive cells in mPFC in two conditioned mice, with(left) or without(right) social expression, respectively. mPFC, medial prefrontal cortex. (b) c-Fos expression in mPFC was more in social group than control as indicated by quantification of the number of the mPFC c-Fos-positive cells (n=12, ***$p < 0.001$, Mann-Whitney test, two tailed). Bars represent mean ± s.e.m. (c) Representative images of c-Fos-positive cells in ACC in two conditioned mice, with(left) or without(right) social expression, respectively. ACC, anterior cingulate cortex. (d) c-Fos expression in ACC was more in social group than control as indicated by quantification of the number of the ACC c-Fos-positive cells(n=12, ***$p < 0.001$, Mann-Whitney test, two tailed). Bars represent mean ± s.e.m. (e) Representative images of c-Fos-positive cells in CA1 in two conditioned mice, with(left) or without(right) social expression, respectively. (f) c-Fos expression in CA1 was more in social group than control as indicated by quantification of the number of the CA1 c-Fos-positive cells (n=12, ***$p < 0.001$, Mann-Whitney test, two tailed). Bars represent mean ± s.e.m.

Supplementary Fig. 5

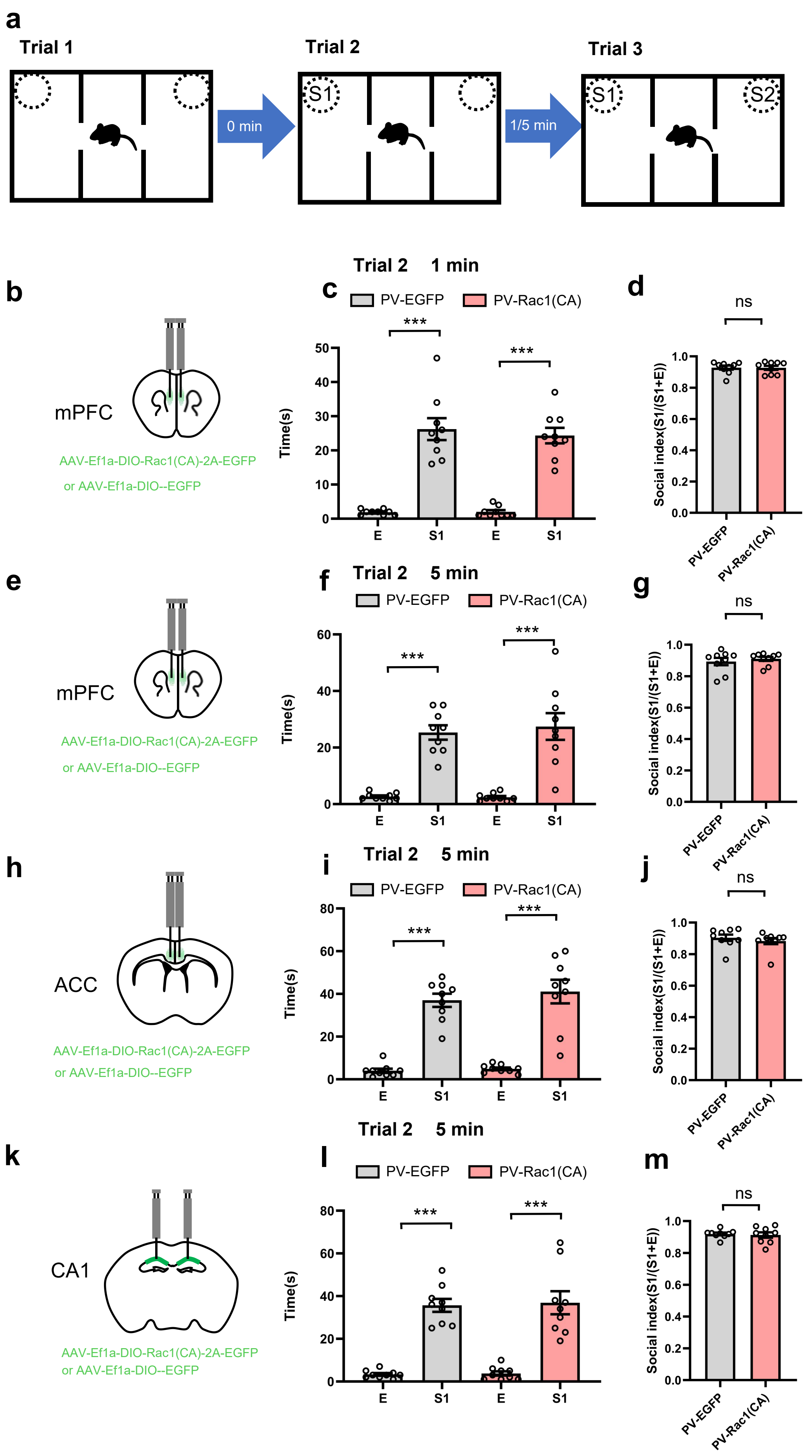

Supplementary Fig. 5: Rac1(CA) in PV neurons of the mPFC, ACC and CA1 has no effect on sociability. (a) Experimental design for social memory using three-chamber sociability and social memory test, for which we designed the inter-trial intervals (ITI) at either 1 or 5 min before trial 3. (b) Location of the virus infection, expresssing Rac1(CA) or EGFP in PV neurons of mPFC. (c-d) When ITI was set at 1 min, PV-EGFP and PV-Rac1(CA) mice showed comparable sociability to stranger 1 (S1) mouse in trial 2 as indicated by more sniff time to S1 mouse than to empty wire cup (E) (n = 9/per group; S1 vs E, ****p* < 0.001, Kruskal-Wallis test) and similar social index among the groups (n = 9/per group, *p* = 0.880. Mann Whitney test, two tailed), suggesting that constitutive activation of Rac1 in PV neurons of mPFC had no effect on sociability. (e) Location of the virus infection, expresssing Rac1(CA) or EGFP in PV neurons of mPFC. (f-g) When ITI was set at 5 min, PV-EGFP and PV-Rac1(CA) mice showed comparable sociability to stranger 1 (S1) mouse in trial 2 as indicated by more sniff time to S1 mouse than to empty wire cup (E) (n = 9/per group; $F_{(3, 13)}$ = 25.940; S1 vs E, ****p* < 0.001, Brown Forsythe ANOVA) and similar social index among the groups (n = 9/per group, *p* = 0.779. Mann Whitney test, two tailed), suggesting that constitutive activation of Rac1 in PV neurons of mPFC had no effect on sociability. (h) Location of the virus infection, expresssing Rac1(CA) or EGFP in PV neurons of ACC. (i-j) When ITI was set at 5 min, PV-EGFP and PV-Rac1(CA) mice showed comparable sociability to stranger 1 (S1) mouse in trial 2 as indicated by more sniff time to S1 mouse than to empty wire cup (E) (n = 9/per group; S1 vs E, ****p* < 0.001, Kruskal-Wallis test) and similar social index among the groups (n = 9/per group, *p* = 0.267. Mann Whitney test, two tailed), suggesting that constitutive activation of Rac1 in PV neurons of ACC had no effect on sociability. (k) Location of the virus infection, expresssing Rac1(CA) or EGFP in PV neurons of CA1. (l-m) When ITI was set at 5 min, PV-EGFP and PV-Rac1(CA) mice showed comparable sociability to stranger 1 (S1) mouse in trial 2 as indicated by more sniff time to S1 mouse than to empty wire cup (E) (n = 9/per group; $F_{(3, 13)}$ = 36.29; S1 vs E, ****p* < 0.001, Brown Forsythe ANOVA) and similar social index among the groups (n = 9/per group, *p* = 0.738. unpaired t test, two tailed), suggesting that constitutive activation of Rac1 in PV neurons of CA1 had no effect on sociability.

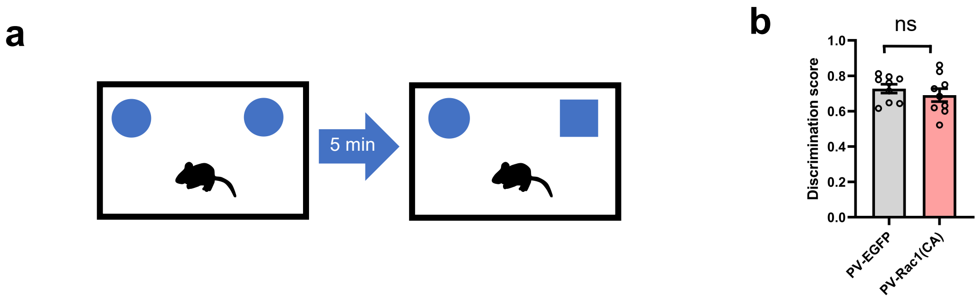

Supplementary Fig. 6: Rac1(CA) in PV neurons of the mPFC has no effect on object memory. (a) Experimental design for object recognition memory with the inter-trial intervals (ITIs) at 5 min between learning and test trial. (b) Rapid forgetting of object memory didn't occur as indicated by that object memory was comparable in both PV-EGFP and PV-Rac1(CA) mice (n=9, p = 0.421, unpaired t test, two-tailed), suggesting that constitutive activation of Rac1 in PV neurons of mPFC had no effect on object memory. Bars represent mean ± s.e.m.

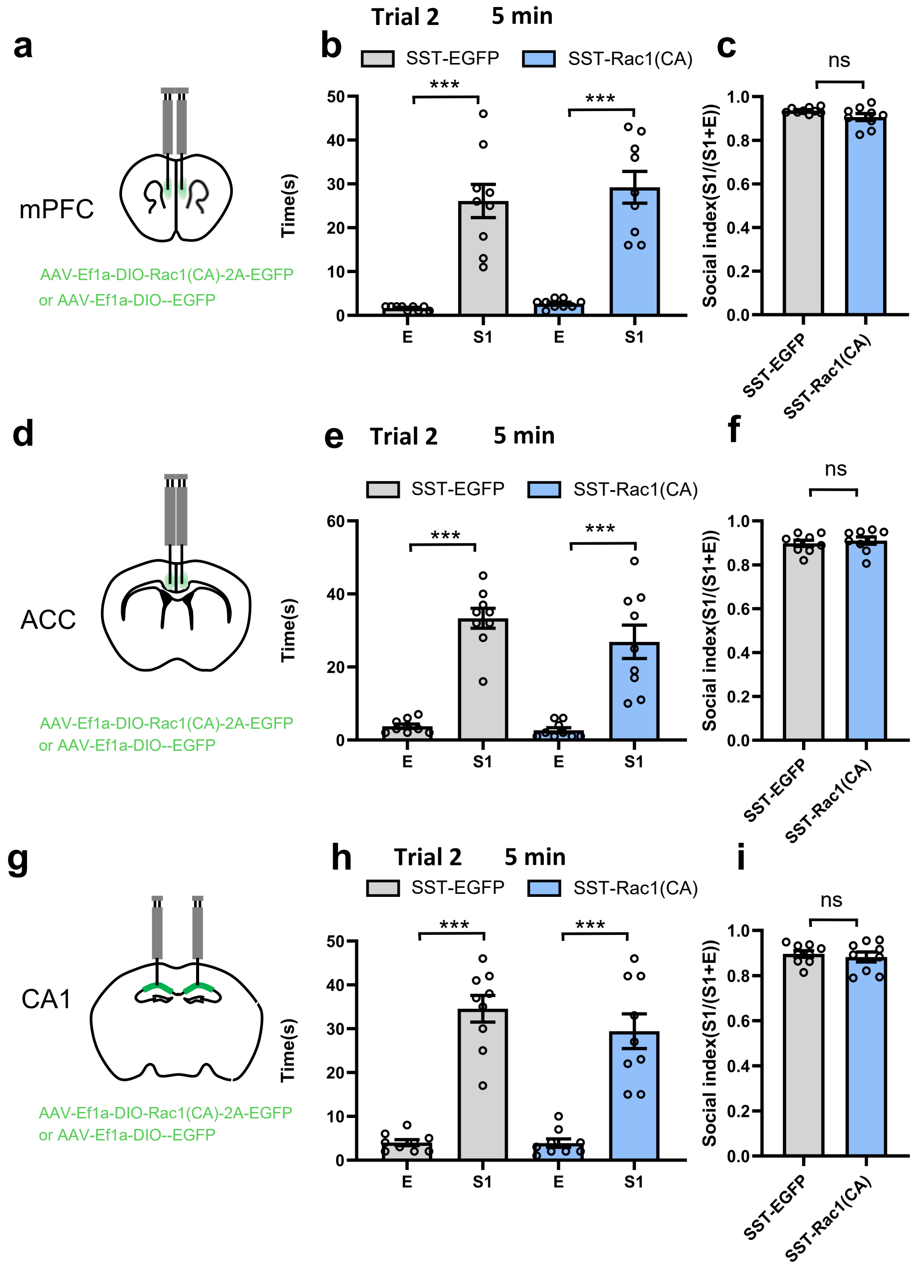

Supplementary Fig. 7: Rac1(CA) in SST neurons of the mPFC, ACC, and CA1 has no effect on sociability. (a) Location of the virus infection, expresssing Rac1(CA) or EGFP in SST neurons of mPFC. (b-c) When ITI was 5 min, SST-EGFP and SST-Rac1(CA) mice showed comparable sociability to stranger 1 (S1) mouse in trial 2 as indicated by more sniff time to S1 mouse than to empty wire cup (E) (n = 9/per group; S1 vs E, ***$p < 0.001$, Kruskal-Wallis test) and similar social index among the groups (n = 9/per group, $p = 0.092$, unpaired t test, two tailed), suggesting that constitutive activation of Rac1 in SST neurons of mPFC had no effect on sociability. (d) Location of the virus infection, expresssing Rac1(CA) or EGFP in SST neurons of mPFC. (e-f) When ITI was 5 min, SST-EGFP and SST-Rac1(CA) mice showed comparable sociability to stranger 1 (S1) mouse in trial 2 as indicated by more sniff time to S1 mouse than to empty wire cup (E) (n = 9/per group; S1 vs E, ***$p < 0.001$, Kruskal-Wallis test) and similar social index among the groups (n = 9/per group, $p = 0.566$, unpaired t test, two tailed), suggesting that constitutive activation of Rac1 in SST neurons of ACC had no effect on sociability. (g) Location of the virus infection, expresssing Rac1(CA) or EGFP in SST neurons of mPFC.(h-i) When ITI was 5 min, SST-EGFP and SST-Rac1(CA) mice showed comparable sociability to stranger 1 (S1) mouse in trial 2 as indicated by more sniff time to S1 mouse than to empty wire cup (E) (n = 9/per group; $F_{(3, 17)} = 40.660$; S1 vs E, ***$p < 0.001$, Brown Forsythe ANOVA) and similar social index among the groups (n = 9/per group, $p = 0.610$, unpaired t test, two tailed), suggesting that constitutive activation of Rac1 in SST neurons of CA1 had no effect on sociability.

Supplementary Fig. 8

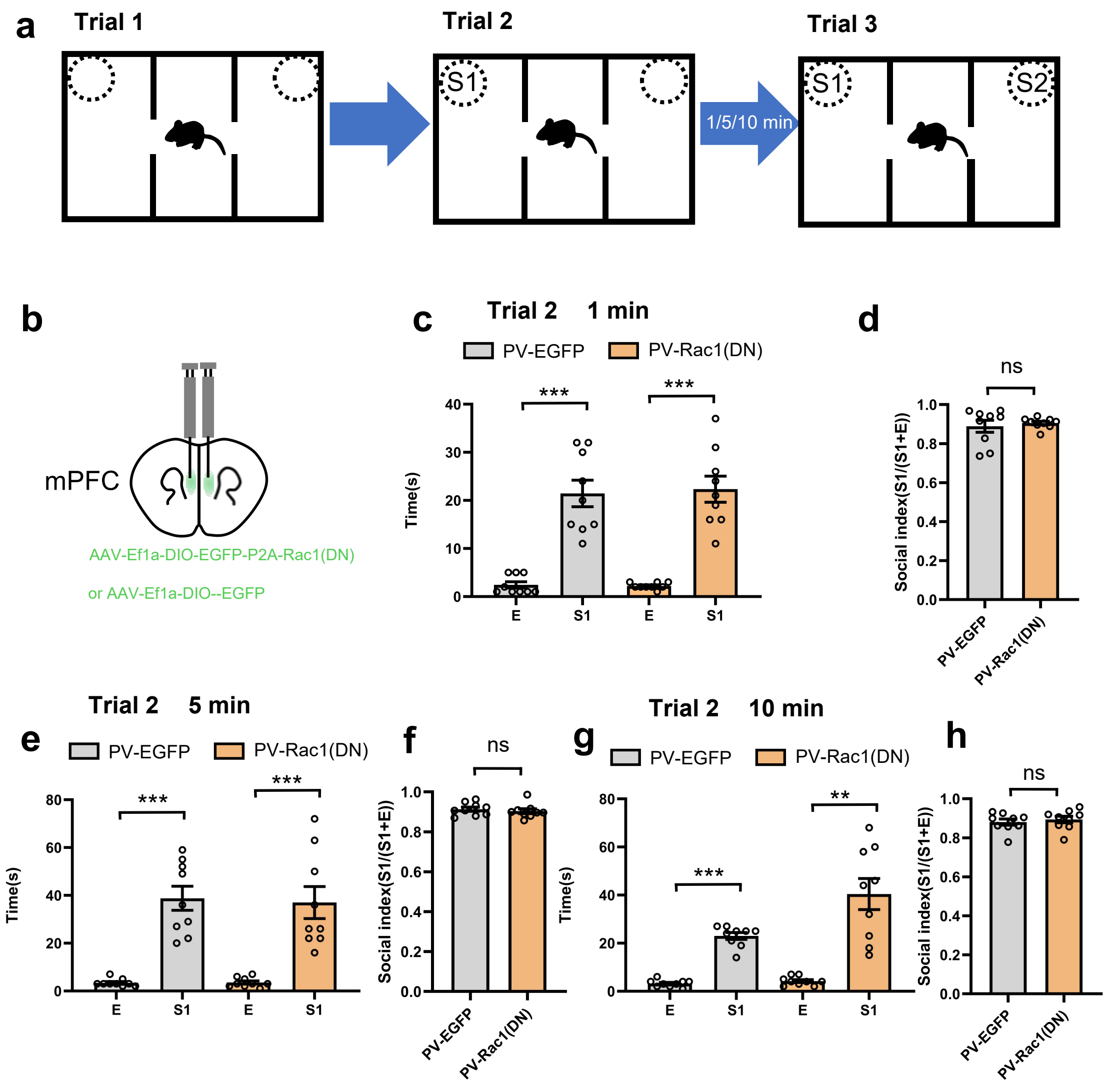

Supplementary Fig. 8: Rac1(CA) in PV neuron of the mPFC has no effect on sociability. (a) Experimental design for social memory using three-chamber sociability and social memory test, for which we designed the inter-trial intervals (ITI) at either 1, 5, or 10 min before trial 3. (b) Location of the virus infection, expresssing Rac1(DN) or EGFP in PV neurons of mPFC.(c-d) When ITI was set at 1 min, PV-EGFP and PV-Rac1(DN) mice showed comparable sociability to stranger 1 (S1) mouse in trial 2 as indicated by more sniff time to S1 mouse than to empty wire cup (E) (n = 9/per group; S1 vs E, ***$p < 0.001$, Kruskal-Wallis test) and similar social index among the groups (n = 9/per group, $p = 0.450$, Mann Whitney test, two tailed), suggesting that inhibition of Rac1 in PV neurons of mPFC had no effect on sociability. (e-f) When ITI was set at 5 min, PV-EGFP and PV-Rac1(DN) mice showed comparable sociability to stranger 1 (S1) mouse in trial 2 as indicated by more sniff time to S1 mouse than to empty wire cup (E) (n = 9/per group; S1 vs E, ***$p < 0.001$, Kruskal-Wallis test) and similar social index among the groups (n = 9/per group, $p = 0.548$, unpaired t test, two tailed), suggesting that inhibition of Rac1 in PV neurons of mPFC had no effect on sociability. (g-h) When ITI was set at 10 min, PV-EGFP and PV-Rac1(DN) mice showed comparable sociability to stranger 1 (S1) mouse in trial 2 as indicated by more sniff time to S1 mouse than to empty wire cup (E) (n = 9/per group; $F_{(3, 9)} = 28.000$; S1 vs E, **$p = 0.001$, ***$p < 0.001$, Brown Forsythe ANOVA) and similar social index among the groups (n = 9/per group, $p = 0.573$, unpaired t test, two tailed), suggesting that inhibition of Rac1 in PV neurons of mPFC had no effect on sociability.

Supplementary Fig. 9

a

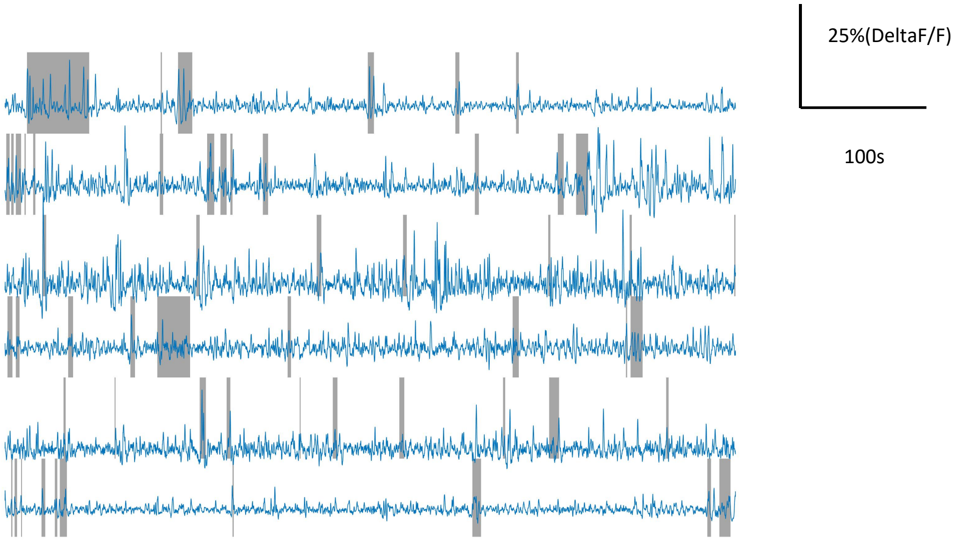

b

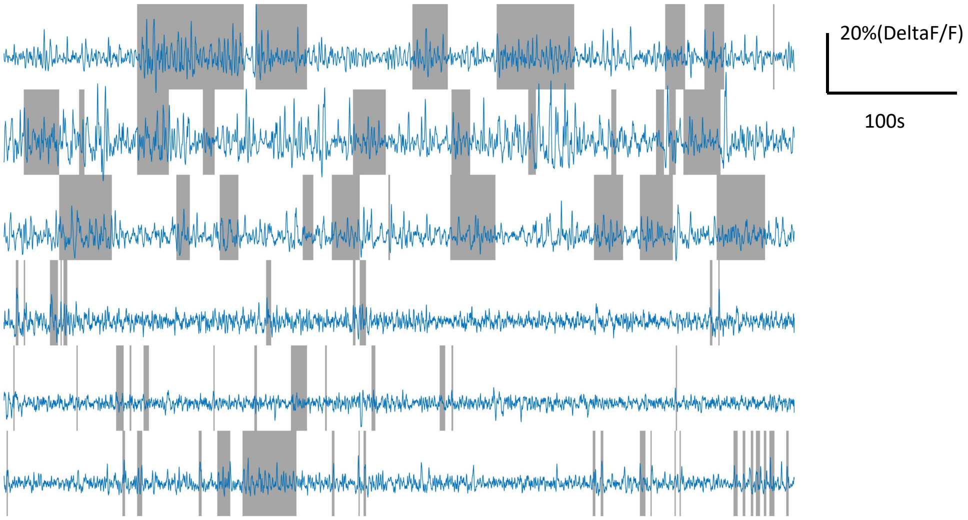

Supplementary Fig. 9: Calcium signals of PV or SST neurons during social behavior. (a) Ca^{2+} signals of PV neurons associated with social behavior in each mouse. Each row represents one mouse, and a total of 6 mice are illustrated. Blue lines indicate the Ca^{2+} signals of each mouse and grey areas indicate social episodes. (b) Ca^{2+} signals of SST neurons associated with social behavior in each mouse. Each row represents one mouse, and a total of 6 mice are illustrated. Blue lines indicate the Ca^{2+} signals of each mouse and grey areas indicate social episodes.

Supplementary Fig. 10

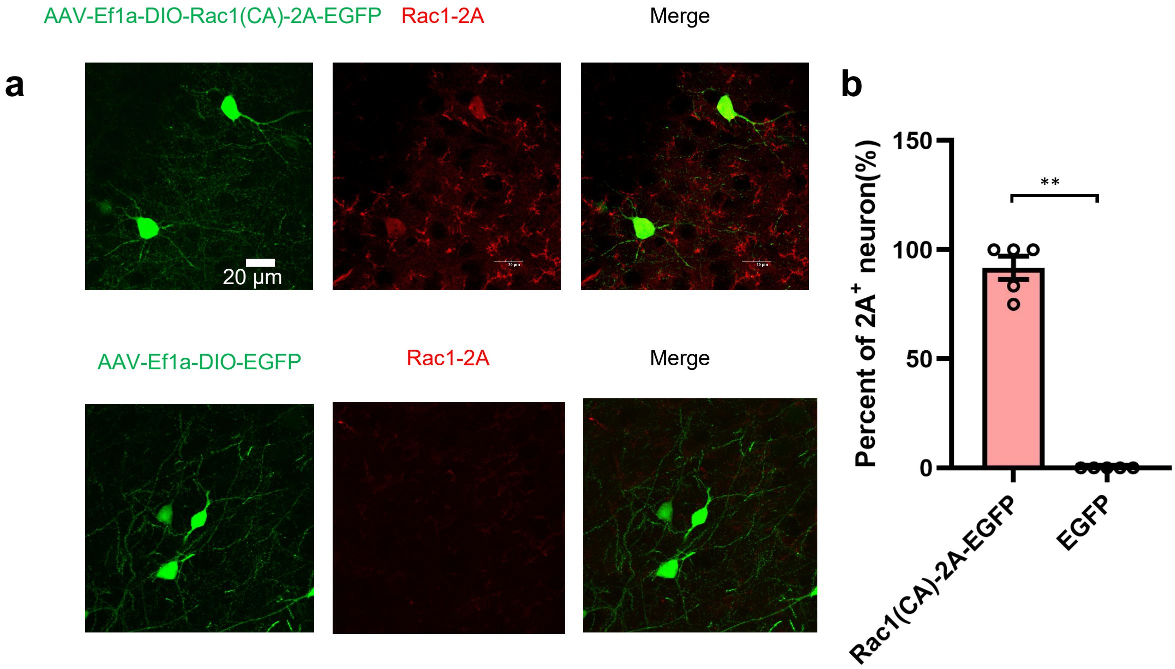

Supplementary Fig. 10: Representative images confirming the expression of AAV-mediated Rac1(CA) in PV neurons of mPFC. (a) Compared to the control group, Rac1(CA) was fused with 2A peptide and the detection of 2A peptide indicated the expression of Rac1(CA) (Red signal). The EGFP signal of AAV is co-labeled with Rac1(CA) (red signal), indicating the successful expression of Rac1(CA) in PV neurons of mPFC. (b) In PV-Cre mice expressing AAV-Ef1a-DIO-Rac1(CA)-2A-EGFP or AAV-Ef1a-DIO-EGFP, percent of 2A positive neuron was calculated by $2A^{+}EGFP^{+}/EGFP^{+}$ (n = 5/per group, $^{**}p = 0.008$, Mann Whitney, two-tailed) .

Supplementary Fig. 11

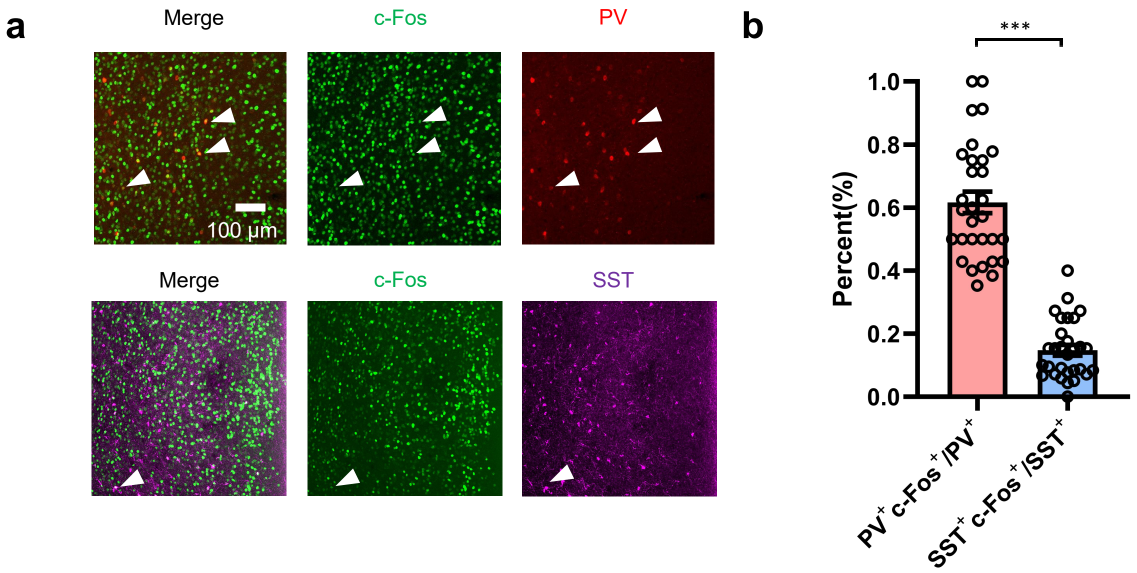

Supplementary Fig. 11: More PV neurons are activated than SST neurons 1h after social memory formation. The number of c-Fos positive neurons were significantly more in PV neurons than SST neurons. (a) Representative images of immunofluorecence of c-Fos, PV, or SST neurons 1 h after social memory formation. (b) The number of c-Fos positive neurons were significantly more in PV neurons than SST neurons 1 h after social memory formation (n=30/per group, ***$p < 0.001$, Mann-Whitney test, two-tailed).

Supplementary Fig. 12

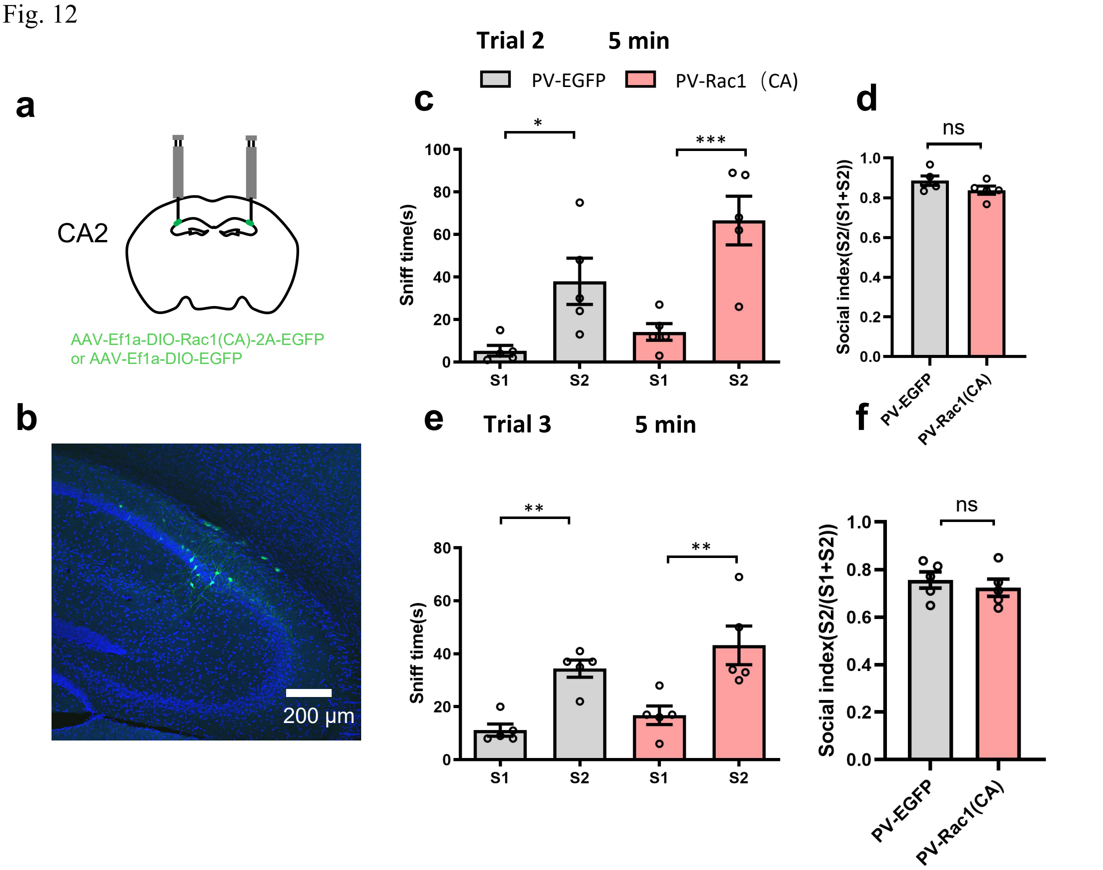

Supplementary Fig. 12: Activating Rac1 in PV neurons of CA2 had no effects on forgetting of social memory. (a) Location of the virus infection, expresssing Rac1(CA) or EGFP in PV neurons of CA2. (b) Representative image of virus expression, expressing Rac1(CA) or EGFP in PV neurons of CA2. (c-d) When ITI was set at 5 min, PV-EGFP and PV-Rac1(CA) mice showed comparable sociability to stranger 1 (S1) mouse in trial 2 as indicated by more sniff time to S1 mouse than to empty wire cup (E) (n = 5/per group; $F_{(3, 16)} = 11.1$; S1 vs E, $p = 0.026$, ***$p < 0.001$, one-way ANOVA) and similar social index among the groups (n = 9/per group, $p = 0.302$, unpaired t test, two tailed), suggesting that activating Rac1 in PV neurons of CA2 had no effect on sociability. (e-f) When ITI was set at 5 min, social preference for the S2 mouse as indicated by more sniff time to S2 than to the S1 mouse was significant both in control (PV-EGFP) and PV-Rac1(CA) mice (n = 9/per group; $F_{(3, 16)} = 10.94$; S1 vs S2, **$p = 0.005$, **$p = 0.001$, one-way ANOVA) and similar social index among the groups (n = 9/per group, $p = 0.580$, unpaired t test, two tailed), suggesting that constitutive activation of Rac1 in PV neurons of CA2 had no effect on social memory.

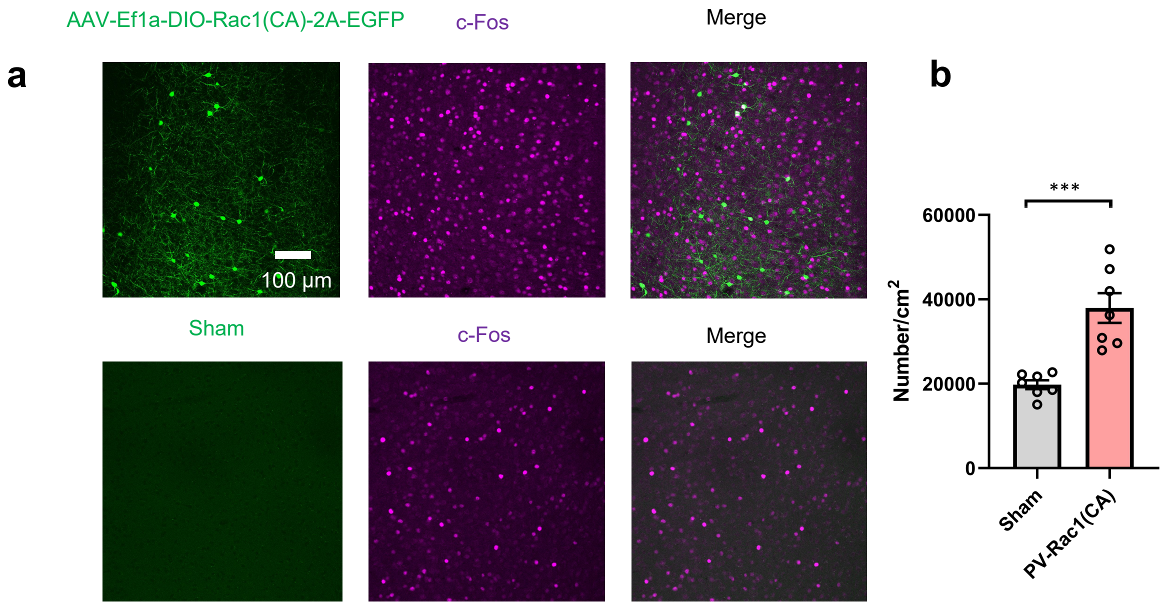

Supplementary Fig. 13: After activating Rac1 in PV neurons of mPFC, the c-Fos expression increased. (a) Representative images of immunofluorecence of c-Fos expression in the PV-Rac1(CA) group and sham injection group. (b) The number of c-Fos positive neurons were more in the PV-Rac1(CA) group than the sham group (n=7/per group, ****p* < 0.001, unpaired t-test, two-tailed).

Supplementary Fig. 14

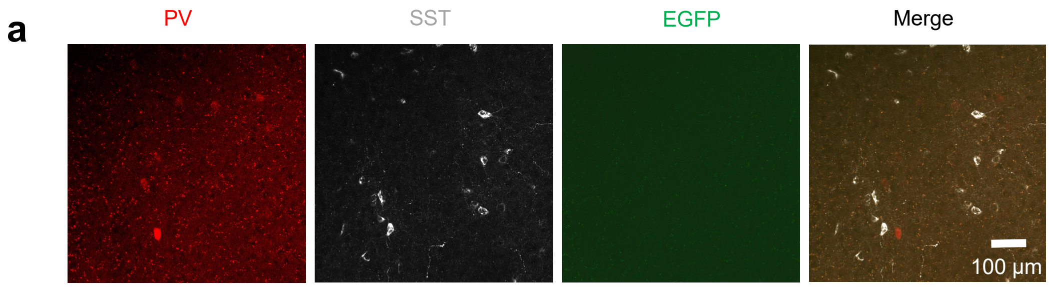

Supplementary Fig. 14: There was no leaky EGFP in Rac1-loxp mice. (a) No leaky EGFP was observed in PV, SST, or other neurons in Rac1-loxp mice.

Supplementary Fig. 15

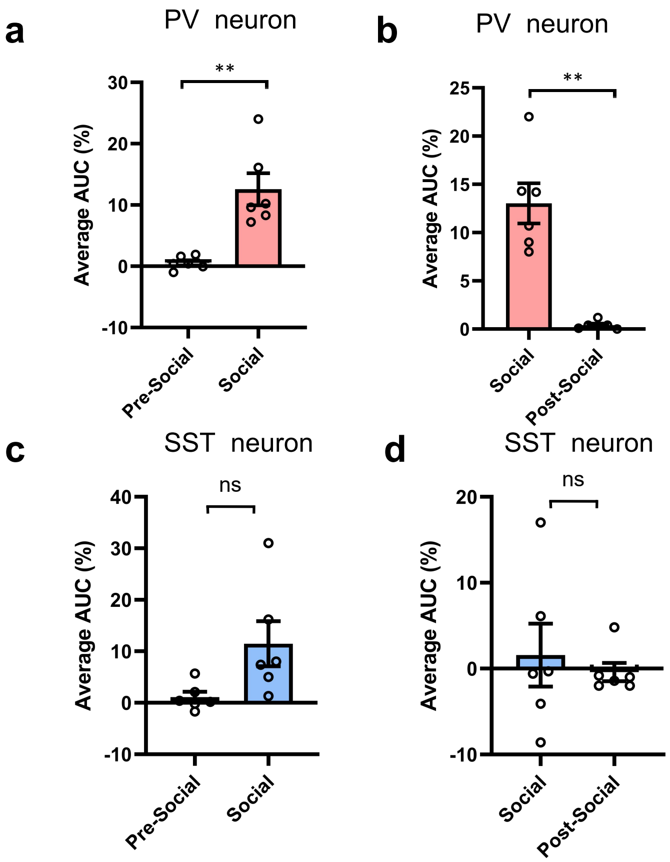

Supplementary Fig. 15: The average AUC of calcium signals in PV or SST neurons respond to the social stimuli. (a) The average AUC of PV neurons was higher in social than in pre-social period (n=6/per group, **p=0.004, paired t test, two-tailed). (b) The average AUC of PV neurons was higher in social than in post-social period (n=6/per group, **p=0.001, paired t test, two-tailed). (c) The average AUC of SST neurons was comparable in social and in pre-social period (n=6/per group, p=0.068, paired t test, two-tailed). (d) The average AUC of SST neurons was comparable in social and post-social period (n=6/per group, p=0.563, Wilcoxon test, two-tailed).
